# Potentially Massive and Global Non-Pyrogenic Production of Condensed “Black” Carbon through Biomass Oxidation

**DOI:** 10.1021/acs.est.3c05448

**Published:** 2024-01-31

**Authors:** Aleksandar I. Goranov, Hongmei Chen, Jianshu Duan, Satish C. B. Myneni, Patrick G. Hatcher

**Affiliations:** † Department of Chemistry and Biochemistry, Old Dominion University, Norfolk, Virginia 23529 United States; ‡ Department of Geosciences, Princeton University, Princeton, New Jersey 08544 United States

**Keywords:** black carbon, condensed aromatic carbon (ConAC), global carbon cycle

## Abstract

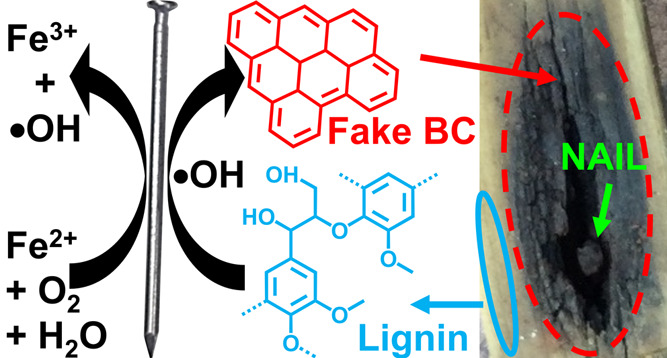

With the increased occurrences of wildfires worldwide, there has been an increase in scientific interest surrounding the chemistry of fire-derived “black” carbon (BC). Traditionally, wildfire research has assumed that condensed aromatic carbon (ConAC) is *exclusively* produced via combustion, and thus, ConAC is equated to BC. However, the lack of correlations between ConAC in soils or rivers and wildfire history suggests that ConAC may be produced non-pyrogenically. Here, we show quantitative evidence that this occurs during the oxidation of biomass with environmentally ubiquitous hydroxyl radicals. Pine wood boards exposed to iron nails and natural weather conditions for 12 years yielded a charcoal-like ConAC-rich material. ConAC was also produced during laboratory oxidations of pine, maple, and brown-rotted oak woods, as well as algae, corn root, and tree bark. Back-of-the-envelope calculations suggest that biomass oxidation could be producing massive non-pyrogenic ConAC fluxes to terrestrial and aquatic environments. These estimates (e.g., 163–182 Tg-ConAC/year to soils) are much higher than the estimated pyrogenic “BC” fluxes (e.g., 128 Tg-ConAC/year to soils) implying that environmental ConAC is primarily non-pyrogenic. This novel perspective suggests that wildfire research trajectories should shift to assessing non-pyrogenic ConAC sources and fluxes, developing new methods for quantifying true BC, and establishing a new view of ConAC as an intermediate species in the biogeochemical processing of biomass during soil humification, aquatic photochemistry, microbial degradation, or mineral–organic matter interactions. We also advise against using BC or pyrogenic carbon (pyC) terminologies for ConAC measured in environmental matrices, unless a pyrogenic source can be confidently assigned.

## Abstract 2

Condensed aromatic carbon (ConAC) has been historically considered to be *exclusively* produced by combustion (e.g., in wildfires). We report on the production of ConAC from biomass oxidation, a non-pyrogenic process that could be producing massive global ConAC fluxes.

## Introduction

Black carbon (BC) is commonly defined as the residue left after the incomplete combustion of biomass during wildfires or other pyrolytic processes (e.g., fossil fuel combustion, biochar production).^1^ Chemically, black carbon is mainly composed of condensed aromatic carbon (ConAC). There are numerous studies that report the formation of ConAC in wildfires^2−4^ and ConAC’s subsequent redistribution in terrestrial (e.g., soil),^5−9^ atmospheric,^10,11^ and fluvial environments^12^ showing that ConAC is globally ubiquitous. As it is viewed that ConAC is equivalent to BC, annual pyrogenic inputs (i.e., of BC) to soils are estimated to be 128 ± 84 Tg-C/year.^13,14^ Hydrologic events, such as rain, mobilize ConAC through river or groundwater systems into the world’s oceans. Riverine pyrogenic fluxes (i.e., of dissolved BC) are estimated to be 18 ± 4 Tg-C/year.^13,14^ Thus, a current paradigm is that wildfires supplying BC (in the form of ConAC) to the environment is a critical process in the global carbon cycle.

Though wildfire research has been ongoing for over two decades, a key fundamental concept remains enigmatic: that ConAC can be used as a proxy for wildfire history. It is currently assumed that ConAC is *exclusively* of pyrogenic origin and thus, scientists equate ConAC to BC. However, studies have shown that there is no correlation between recent fire events and ConAC in soils^15^ or dissolved ConAC (dConAC) in freshwaters.^16,17^ Interestingly, strong correlations have been observed between ConAC and soil organic carbon (SOC) in terrestrial systems,^8,18−22^ as well as between dConAC and dissolved organic carbon (DOC) in aquatic systems.^13,23^ These correlations exist on various spatial and temporal levels, even in systems with no recent wildifre exposure,^24,25^ which suggests that the production of ConAC and dConAC is coupled to the production of SOC and DOC, hinting that the existence of ConAC and dConAC occurs independently of combustion.

Recent reports suggest that ConAC can be a by-product of the oxidation of lignin, the second most abundant biopolymer on Earth. Biomass oxidation is a natural process involved in the formation of soil but also occurs during the export of terrestrial organic matter into the ocean (via photochemical or other oxidative pathways). Non-pyrogenic ConAC formation was first proposed in experimental studies^26,27^ in which reactive oxygen species (ROS), such as hydroxyl radicals, attacked lignin to polymerize it into ConAC. ROS can be generated abiotically either via photochemistry or by the dark Fenton reaction.^28,29^ Another recent study observed non-pyrogenic formation of ConAC in the aerobic microbial incubation of wheat straw,^30^ an example of a microbiological system where microbes exude extracellular enzymes, which release ROS. These mechanistic studies explain the observation of non-pyrogenic ConAC formation during soil humification^31^ and photochemical DOC irradiation.^26^ However, due to the use of qualitative (electrospray ionization–mass spectrometry) or less conventional methods (spectral editing nuclear magnetic resonance (NMR) spectroscopy) in these studies, their results “warrant further investigation using quantitative methods such as the benzenepolycarboxylic acids (BPCA) method [sic]”.^12^

Here, we present quantitative evidence that ConAC can be formed non-pyrogenically during biomass oxidation. We illustrate this by using the Fenton reaction to produce hydroxyl radicals, a type of ROS. This reaction involves iron (Fe), a highly ubiquitous element in the environment (e.g., up to 55% in soils).^32^ Fe-driven oxidation occurs globally and is heavily involved in the chemical transformation of organic matter in soils, groundwater, and surficial aquatic systems.^33^ Furthermore, hydroxyl radicals are common ROS for many other processes, including photochemistry,^34−36^ biomass decomposition,^37,38^ primary productivity,^39−41^ and oxidation driven by other metals (e.g., manganese).^42,43^ Thus, the results of this study can be applied globally without having the requirement to have high concentrations of Fe.

**Figure 1:**
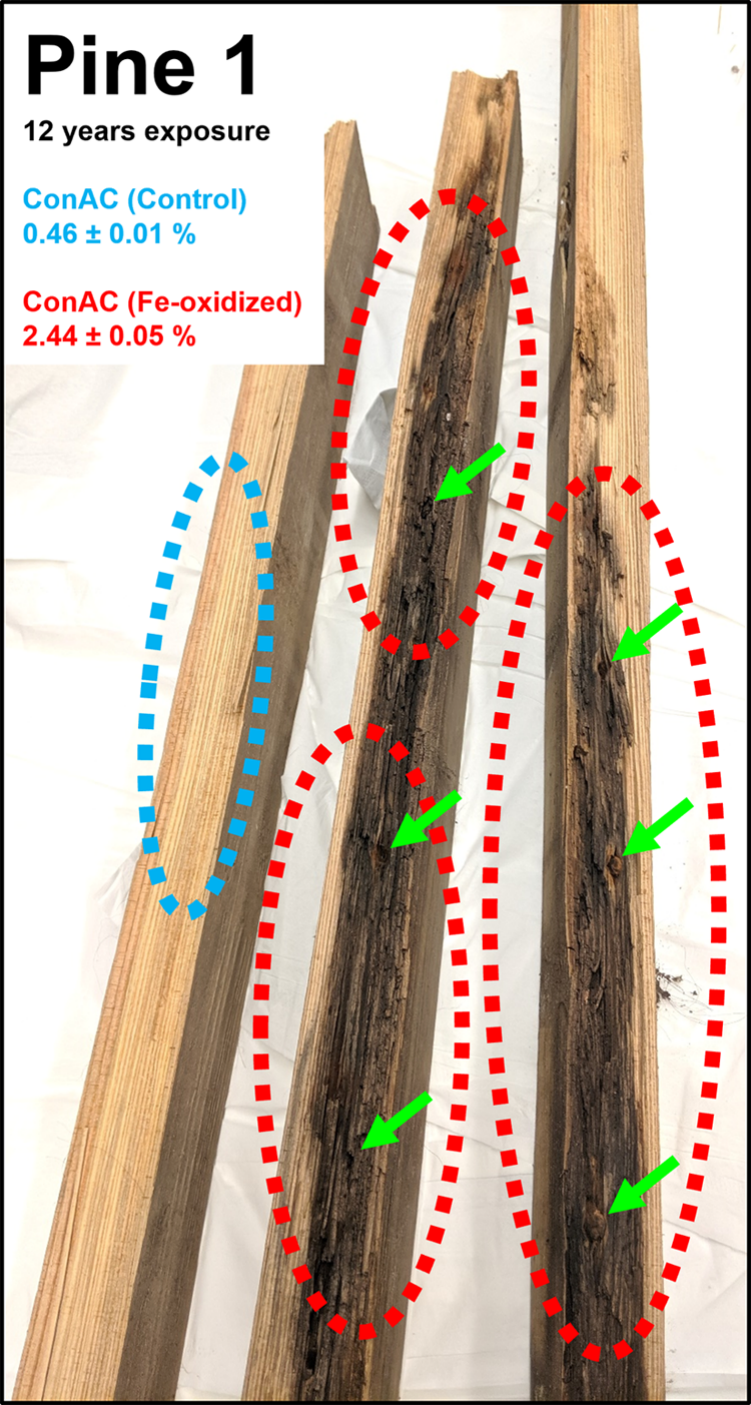
Charcoalification of fresh pine wood boards through exposure to Fe nails. The blue circle indicates a zone with no visible charcoalification, which was sampled to represent the control. The red circles indicate zones with clear non-pyrogenic charcoalification. The green arrows show Fe nails embedded in the wood planks. ConAC quantities relative to organic carbon, as well as lengths of exposure to nails, are shown in the legend. Adapted with permission from Goranov.^45^ Copyright 2020 Aleksandar Ivaylov Goranov, All Rights Reserved.

To study the products of environmental oxidation, we examined three wood boards that were weathered by high concentrations of Fe. Two different deck constructions generated two pine wood boards that were assembled with Fe nails 12 years ago and were exposed to natural wetting and drying events. Upon recent dismantling of the two decks, the obtained boards were observed to have undergone a charring-like process (“charcoalification”) emanating from the Fe nails used to connect the boards and in contact with cross wood pieces on the underside that was not exposed to sunlight (Figures 1 and S1). A third specimen of a maple wood board had been nailed as part of a pallet and exposed to the weather for a period of one year, and a charring-like process was also observed (Figure S1). These wood-nail systems can be viewed as models that conceivably mimic the exposure of biomass to ROS. The nails provide a source of iron (Fe^0^), which is rapidly oxidized (“rusted”) to Fe^II^ or Fe^III^. The Fe^II^ can then participate in the Fenton reaction, during which oxygenated water from rain reacts with Fe^II^ to produce ROS^28,29,44^ that then react with the organic matter from the wood. We show that ConAC is produced in this process, which contributed to the darkening of the woods in addition to the production of Fe^III^ compounds. To obtain causal proof for the increase in ConAC in the darkened parts of the woods, we also performed laboratory Fenton oxidations of different woody (pine, maple, and brown-rotted oak) and non-woody biomass (corn root, bark, and algae).

We propose that non-pyrogenic charcoalification can be widespread in the environment, particularly in systems where Fe-bearing minerals intermixed with SOC are undergoing wetting and drying cycles, or in aquatic systems where DOC undergoes ROS-driven oxidation (e.g., photodegradation, microbial degradation). It is a globally transformative revelation, as we demonstrate that ConAC can be produced during the oxidation of different biomass materials that have not experienced combustion. Our quantitative proof of the existence of this process requires the development of not only revised estimates for pyrogenic sources of ConAC in various environmental systems but also a new understanding for ConAC as an intermediate in biomass degradation processes rather than a group of compounds specifically derived from wildfires or other thermogenic processes. The BC and pyrogenic carbon (pyC) terminologies and commonly used analytical methods in the wildfire sciences also need to be revisited.

## Materials and Methods

### Samples

A pine wood specimen (Pine 1) was collected from a deck construction of a house in Norfolk, VA. An entirely different pine wood specimen (Pine 2) was collected from a deck construction of a house in Suffolk, VA. Pine 2 was of pressure-treated wood (retarding microbial degradation), whereas Pine 1 was untreated with no visible signs of decay. The owners of the two houses provided statements that their decks have never been struck by lightning, which excludes the possibility of electricity-driven “Lichtenberg” burning.^46^ A third specimen of sugar maple (Maple, *Acer saccharum)* was held as part of a pallet exposed to the elements for a year.

The three wood specimens were rinsed with ultrapure water (18.2 MΩ) and air-dried under cover. Charcoalified wood pieces from the areas near the nails were sampled with precombusted tweezers. Wood samples remote from the nails were sampled using a precombusted wood scraper. Sampling was done from numerous “control” and “Fe-oxidized” zones (Figures 1 and S1) to obtain representative samples. All samples were ground and sieved to fine powders (Mesh #80, 0.177 mm opening).

An algae sample containing species of the *Scenedesmus* and *Desmodesmus* genera was obtained from the algal raceway water at the Old Dominion University algae farm (Spring Grove, VA).^47^ The algal biomass was used to represent microbial biomass, which would exist across various terrestrial and aquatic environments. The bark of Yellow Birch trees (*Betula alleghaniensis*), located in Blacksburg, VA, was gently peeled and collected as a suberin-rich representative biomass.^48^ Suberin is a biopolymer found in cell walls of phellem, endodermis, exodermis, wound tissues, abscission zones, bundle sheath, and other tissues of plants.^49^ A lignin-rich sample was obtained from White Oak wood (*Quercus alba*) at a mixed deciduous/pine forested site in Suffolk, VA.^50^ The wood had been infested by brown-rot fungi, which degraded the cellulose, proteins, and lipids without consuming the lignin and thus, this sample had naturally become a lignin concentrate.^51^ This sample was used to test how the ConAC production rate varies when labile materials (carbohydrates, proteins, etc.) are not present. Corn root, representing root biomass, was supplied by Tsutomu Ohno (University of Maine).

### Laboratory Oxidation of Diverse Biomass

To simulate natural oxidation, controlled Fenton experiments were conducted using six types of biomass in prebaked, acid-washed, dark, sealed vials, eliminating the possibility of photochemistry or atmospheric deposition affecting the experiment. ConAC was quantified in the pure biomass materials, providing a baseline for ConAC in each digestion vessel at the start of the reaction. About 30 mg of biomass with predetermined C% and ConAC% were suspended in 20 mL of acidic aqueous solution (pH = 3 from HCl; Fischer Scientific, Certified ACS grade) containing 80 ppm Fe^2+^ (as FeSO_4_; Mallinckrodt Chemicals, ACS grade) and 2 M H_2_O_2_ (Fischer Scientific, Certified ACS grade). Incubations lasted 2 days, which was the approximate time for the near-complete consumption of H_2_O_2_ (no visible bubble formation). One-day incubations were also conducted to obtain an additional time point.

Maple wood was used for a second oxidation experiment, which was sustained over 10 days. This biomass was chosen as it appeared to be most pristine (i.e., had experienced little to no environmental aging). About 30 mg of powdered wood was suspended in 100 mL of acidic aqueous solution (pH = 3) containing 50 ppm Fe^2+^ and 1 M H_2_O_2_. Additional FeSO_4_ and H_2_O_2_ were added at days 2, 4, 8, and 10 to final concentrations of 50 ppm of Fe^2+^ and 1 M H_2_O_2_ to keep the reaction going and sustain a steady-state flux of hydroxyl radicals.

In both experiments, the vials were kept sealed and on a shaker table, allowing for gentle agitation. At each time point, a vial was sacrificed by adding 10 mL of methanol (Fisher Scientific, Optima LC-MS grade) that quenched the Fenton reaction, and the vial was transferred to an −80 °C freezer to prevent further oxidation. The liquid was removed by freeze-drying in order to recover all organic carbon (both particulate and dissolved) in these closed systems. The obtained powder was weighed and analyzed to determine its C% and ConAC% and to compute the amounts of C and ConAC present after the oxidation. Procedural blanks (H_2_O+HCl+FeSO_4_+H_2_O_2_) were analyzed to confirm that there was no extraneous ConAC added to the experimental systems. Any extraneous C was accounted for via blank subtraction.

Biomass-to-ConAC conversion (at time point *t*) was calculated as the ratio of produced ConAC (at time point *t*) during the oxidation relative to the amount of biomass-carbon used in the experiment (i.e., at time point 0). The quantity of produced ConAC is calculated as the amount of ConAC in the sample at time point *t* corrected for the ConAC added from the biomass at time = 0 (eq 1). Note that ConAC quantities here are total ConAC in the systems and are not fractionated as particulate ConAC or dissolved ConAC (dConAC).
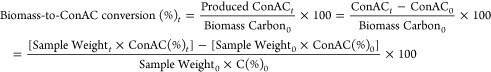
1

### Quantification of ConAC via the Benzenepolycarboxylic acids (BPCA) Method

Dried powdered samples, no more than 5 mg carbon-equivalents,^52^ were weighed in 20 mL glass ampules. Concentrated nitric acid (2 mL, 65% HNO_3_, J.T. Baker, trace metal grade) was added and the ampules were allowed to sit for 15 min.^53^ Then, they were flame-sealed and thermolyzed in a programmable oven for 9 h at 170 °C. After the digestion, the nitric acid was evaporated at 60 °C in a sand bath under a gentle stream of ultrapure N_2_ gas (Airgas, UHP300). The BPCA-containing residue was then dissolved in 2 mL of 0.6 M phosphoric acid and filtered using a 0.2 μm PTFE filter into an autosampler vial. Only benzenehexa- (B6CA) and benzenepentacarboxylic (B5CA) acids were quantified because these markers have been found to be most reliable as being produced only by ConAC.^12,54^ Other markers, such as the benzenetri- and benzenetetracarboxylic acids were not considered as they can be produced after the nitric acid oxidation of ligninaceous molecules.^52,55^ B6CA and B5CA were quantified chromatographically on an Agilent 1100 high-performance liquid chromatography (HPLC) system. Separation was achieved utilizing an organic-free gradient of 0.6 M phosphoric acid (pH = 1) and a phosphate buffer (20 mM, pH = 6) on an Agilent Poroshell 120 Phenyl-Hexyl (4.6 × 150 mm, 2.7 μm) column following published procedures.^53^ Injection volumes were varied from 5 to 30 μL and markers were detected spectrophotometrically at 254 nm and quantified using external calibration curves. The measured quantities of these two biomarkers (in mg BPCA-carbon produced after the oxidation, BPCA_C_) were related to the initial concentration of ConAC in the samples and then normalized to the sample’s organic carbon content (eq 2).^55^ The conversion factor of 7.04 has been developed by quantifying B6CA and B5CA yields after the HNO_3_ oxidation of carbon nanotubes, a standard material made entirely of ConAC.^55^ All BPCA measurements were with relative standard deviations below 5%.
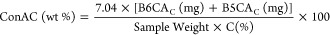
2

## Results and Discussion

### Production of ConAC via Non-Pyrogenic Fenton Oxidation in Model Nail-Wood Systems

To evaluate the chemical changes that have induced visual charcoalification without burning, materials from charcoalified and non-charcoalified locations were obtained for the three wood specimens (Pine 1, Pine 2, and Maple). The blackened solid hereafter is termed an “Fe-oxidized” sample, and wood remote from the nail is termed a “control” sample. Quantitative assay for ConAC using BPCA markers^53^ revealed that ConAC was formed upon exposure to Fe (Table S1). The two Fe-oxidized pine samples (12 years of exposure to nails) contained 5.30 and 4.96 times more ConAC than their corresponding controls (Figures 1 and S1). The presence of tiny, but detectable concentrations of ConAC in the control samples is likely due to Fenton-produced ConAC in the Fe-exposed areas diffusing throughout the boards during the exposure to natural wetting conditions for 12 years, as typically woods are ConAC-free.^55^ Assuming the original board was ConAC-free, oxidation produced 0.195% ConAC per year (note that this is the ConAC that was retained in the wood). This rate was likely much higher as oxidation forms oxygen-containing functional groups (e.g., OH, CHO, COOH) that make ConAC easily solubilizable by rain. This explains why large amounts of material were missing from the areas closer to the nails - the wood oxidation had likely converted some of the carbon to CO_2_, but also rain had likely extracted water-soluble molecules and taken ConAC out of the system as dConAC. The Fe-oxidized Maple contained 1.45 times more ConAC than its control (Figure S1). Thus, over one year, 0.068% ConAC was formed next to the nails in this board.

Collectively, we present strong, quantitative evidence that ConAC can be produced from biomass oxidation, which is a non-pyrogenic process. Because all boards were nailed to an adjacent cross piece, where the nailing kept the wood in the dark, photochemistry was eliminated as being responsible for the oxidative alteration. Portions of the boards remote from the Fe nails appeared to be virtually intact, mainly indicating that microbial decomposition of the wood had been retarded, especially in the case of Pine 2 (Figure S1), which had been chemically treated to retard microbial alteration. Our multi-instrumental analyses revealed that the pressure-treatment agent in Pine 2 did not influence the non-pyrogenic ConAC formation process (see Section 7 in the Supporting Information, SI).

**Figure 2:**
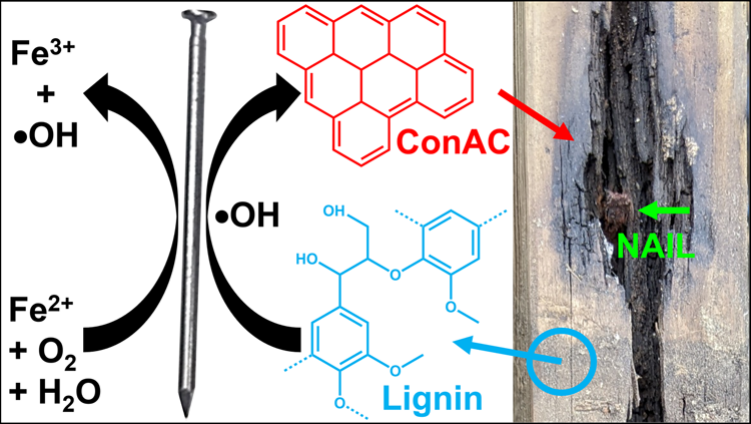
Illustrated production of ConAC (in red) from lignin (in blue) through Fenton chemistry driven by the Fe nail (pointed by the green arrow). The photograph on the right shows one of the charcoalified areas of the Pine 2 sample.

The mechanism of converting biomass to ConAC has been previously proposed by Waggoner.^27^ Briefly, aromatic compounds (in lignin, tannins, or other biopolymers), upon exposure to hydroxyl radicals (produced by the Fenton reaction, photochemistry, or even microbial enzymes) can be oxidized to unsaturated aliphatic and hydroxylated carboxyl-containing compounds. The oxidation products then undergo cyclization via Diels–Alder reactions. The cyclic products, upon exposure to more hydroxyl radicals, can be aromatized to ConAC via hydrogen abstraction. In our study, hydroxyl radicals were produced by the Fenton reaction, and the overall ConAC production is illustrated in Figure 2. This pathway is supported by supplementary characterization with solid-state ^13^C NMR (Section 4 in the SI), ultrahigh-resolution mass spectrometry (Section 5 in the SI), and X-ray absorption near-edge structure spectroscopy (Section 6 of SI). These analyses reveal that the chemistry behind the wood darkening is similar to what is observed in deep soil and sedimentary horizons, i.e., our wood-nail systems simulate the natural process of plant litter degradation, during which soil organic matter is formed (i.e., humification). Thus, ConAC is likely an important intermediate giving soils some of their aromatic character. It must be noted that the aromaticity of soils (and of other types of environmental samples) depends on the concentrations of monoaromatics, polyaromatics, and ConAC. Thus, caution should be exercised to not assume that organic matter aromaticity comes exclusively from ConAC.^56^ While our ancillary characterizations suggest the production of non-condensed aromatics, such as polyphenols (Figures S2 and S4), quantitative methods, such as lignin-phenol quantification,^57^ are needed to discern the extent of which oxidation (via Fenton or other pathways) drives the natural organic matter aromaticity to increase.

### Laboratory Fenton Oxidation of Environmentally Representative Biomass Materials

While the three wood boards clearly show that ConAC was produced from exposure to hydroxyl radicals via Fe nails, we performed controlled laboratory experiments using several different biomass materials to further strengthen our case. The oxidation conditions were set to be harsh (i.e., high concentrations of Fe^2+^ and H_2_O_2_),^27^ since simulating environmental oxidation rates on the time scale of months or years in a laboratory setting is impractical, and the primary goal of these experiments was to determine whether ConAC would be produced upon exposure to Fenton chemistry rather than measuring ConAC production kinetics.

**Figure 3:**
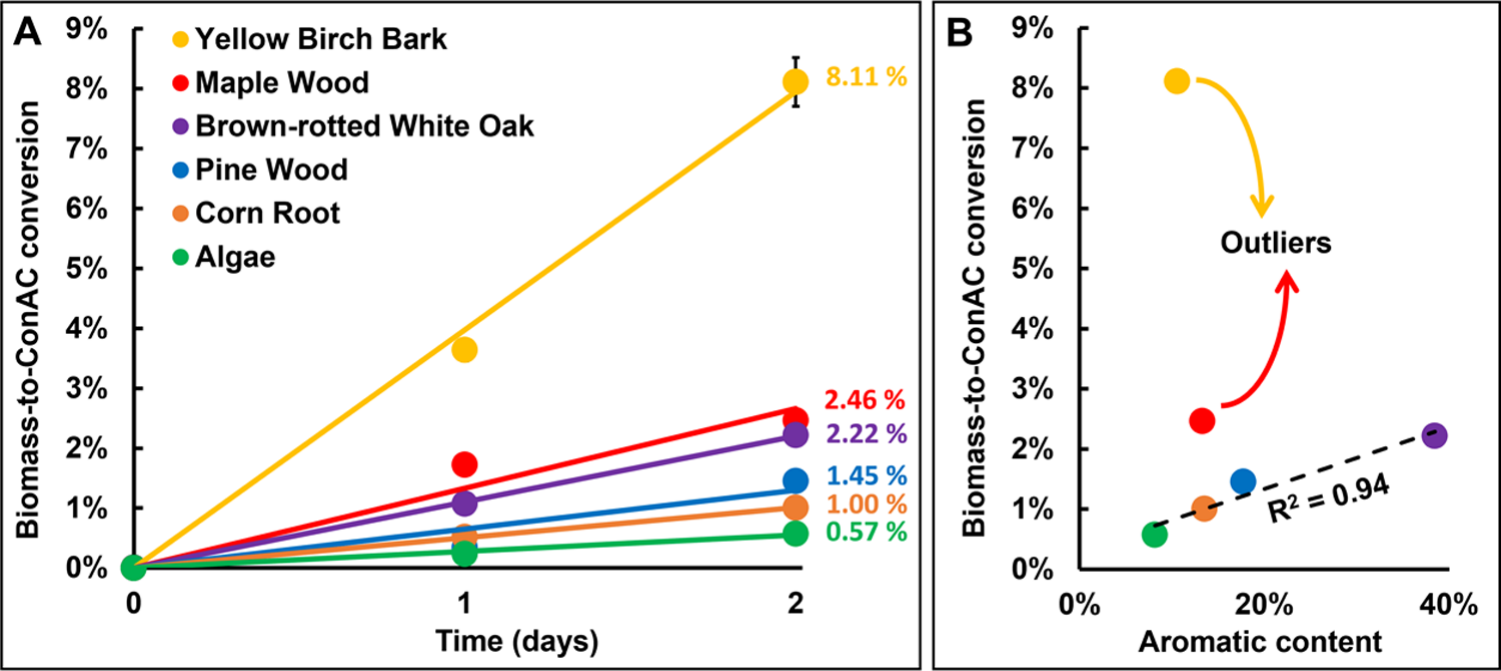
Laboratory oxidations of different biomass materials (A) in which biomass materials were mixed with FeSO_4_ and H_2_O_2_ at the start of the experiment. Produced ConAC quantities were presented relative to the original amount of biomass-carbon, and ConAC present in the starting material was accounted for using a background correction. All measurements have an associated conservative uncertainty of 5% as listed in Table S3. (B) Comparison of the biomass-to-ConAC conversion (at day 2) to the aromatic content of the biomass materials based on their ^13^C NMR spectra (Figure S7).

Quantification of ConAC before (at time = 0) and after oxidation (at time points of 1 or 2 days) revealed that ConAC was produced from all biomass (Figure 3A). The bark had the highest conversion of 8.11% (i.e., 8.11% of the initial biomass-C was converted to ConAC). The bark was followed by the woody materials (maple wood, brown-rotted oak, and pine wood) and then the corn root. The algae biomass was converted to ConAC to the least extent (0.57%). These experiments provided direct causal evidence that Fenton oxidation of biomass produces ConAC. Interestingly, more maple biomass was converted to ConAC (2.46% over 2 days) than pine biomass (1.45%) in the laboratory experiments, whereas the maple board showed a slower ConAC production rate (0.068%/year) than the two pine boards (0.186–0.203%/year, Table S1). This was likely because in the environmentally exposed boards, the oxidation was initially slow as Fe^0^ from the nails had to be oxidized to Fe^II^ in order to initiate the conversion of biomass to ConAC. The ConAC production rate of the Maple would have likely been higher if this board had been actively exposed to the environment over a longer period (e.g., 10+ years).

To assess the cause of differences in biomass-to-ConAC conversion rates (i.e., why did the bark produce so much ConAC while the algae produced so little), we compared the biomass-to-ConAC conversion at day 2 with solid-state NMR data for these samples (Figure S7). The aromatic contents of four of the samples, as determined by the sum of the aryl and phenolic NMR signals, correlated well with the ConAC production rates (Figure 3B). This indicated that the abundance of aromatic compounds likely controlled this process: samples higher in aromatics would produce more ConAC upon exposure to oxidation. For most of the biomass types here, these aromatics correspond to lignin phenols. However, the bark is rich in suberin (an aliphatic polymer containing monoaromatic rings),^48^ and the algae contains aromatic rings in its proteins. This correlation provides an additional validation to our claim that aromatics, such as lignin, can be radically polymerized to ConAC. Other functional groups belonging to carbohydrates and lipids (e.g., aliphatic groups) did not correlate with ConAC production rates suggesting that non-aromatic compounds did not control this non-pyrogenic process. The maple wood and yellow birch bark stood out as outliers. These two samples have similar aromatic content to the corn root sample (∼12%) but clearly had vastly different biomass-to-ConAC conversion rates. Thus, there must be at least one additional, presently unknown, controlling factor in the kinetics of oxidative ConAC production (e.g., presence of natural ROS quenchers or other metals acting as catalysts). Discovering what controls the rate of biomass-to-ConAC conversion should be a priority in future studies. Such factors (e.g., aromatic content of biomass) may be useful predictors for the non-pyrogenic ConAC production rates in different environmental systems. This can allow for modeling global non-pyrogenic ConAC production rates in order to predict accurate BC fluxes and reservoirs in the biogeosphere.

**Figure 4:**
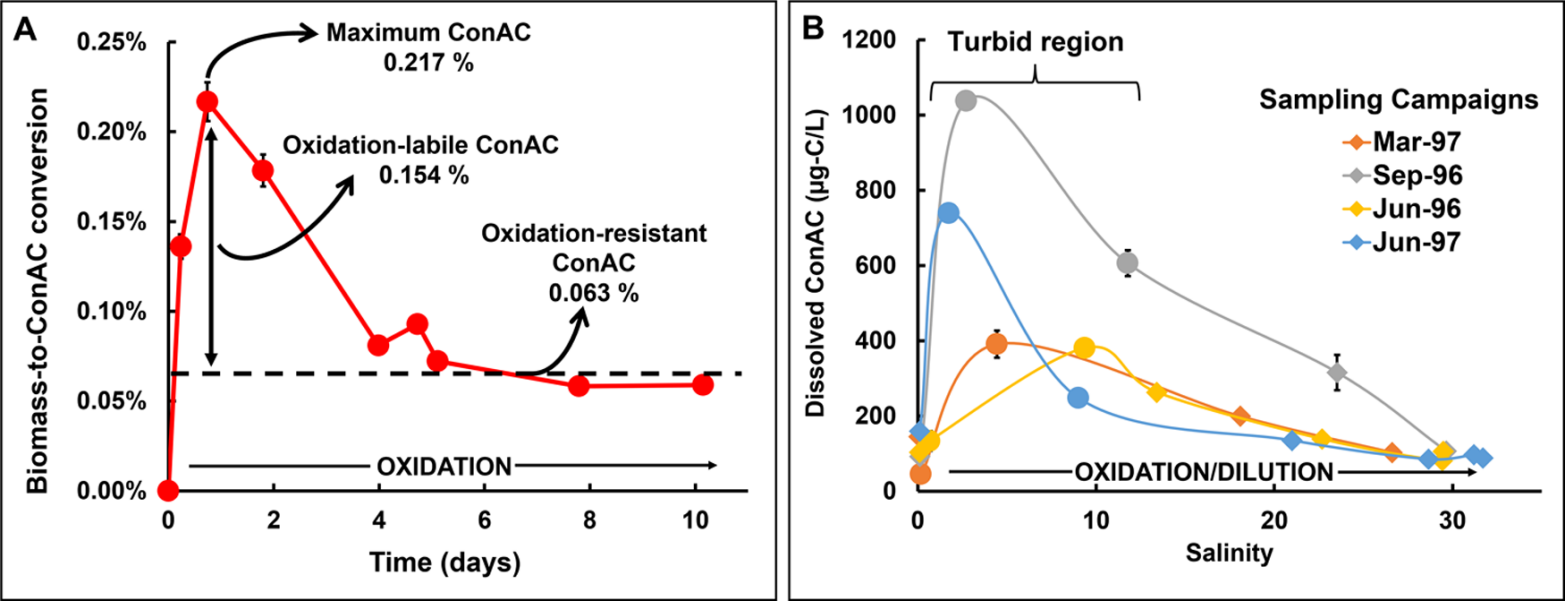
Laboratory oxidation of maple wood (A). Produced ConAC quantities were presented relative to the original amount of biomass-carbon, and ConAC present in the starting material was accounted for using a background correction. All measurements have an associated conservative uncertainty of 5% as listed in Table S4. Note that the maximum biomass-to-ConAC conversion (up to 0.217%) in this experiment was less than that of the harsher experiment (up to 2.46%, Figure 3) due to different FeSO_4_ and H_2_O_2_ concentrations. (B) ConAC profiles along a salinity transect of the Delaware Estuary (chemothermal oxidation ConAC measurements from Mannino and Harvey).^60^

Though these results prove that ConAC is produced by oxidation, ConAC is also known to be labile to oxidation.^58,59^ Upon oxidation, the condensed aromatic rings of ConAC are gradually opened until a pool of aliphatics is formed alongside the production of gases (e.g., CO_2_).^59^ To test if ConAC oxidation would occur or if the ConAC production rate would remain linear, the maple wood was also exposed to a longer, sustained Fenton oxidation experiment that lasted over 10 days (Figure 4A). The biomass-to-ConAC conversion first increased to 0.217 ± 0.011% and then decreased until biomass-to-ConAC conversion reached a stable baseline of 0.063 ± 0.008%. This indicates that ConAC production and degradation occurred simultaneously during the oxidation gradient, making it extremely difficult to quantitatively study non-pyrogenic ConAC formation processes. Interestingly, ConAC exhibits similar behavior across estuary transects (Figure 4B). In such environments, hydrologically mobilized ConAC from land, atmospheric deposition, or release from resuspended sediments cause ConAC to increase at first, but later dilution and oxidative degradation cause ConAC to decrease.^60^ While this is undoubtedly true, biomass upstream (leaching from soils or being generated by primary productivity) could also be converted into ConAC by *in situ* photochemistry or Fenton reactions on mineral surfaces, particularly in the turbid region of the estuary. Such *in situ* production of ConAC would contribute to its increase observed at first and later counteract the degradation/dilution observed downstream.

### Estimation of Non-Pyrogenic ConAC Fluxes to Global Terrestrial and Aquatic Systems

To illustrate the potential impact of our findings to the global BC cycle,^61^ we performed back-of-the-envelope calculations to estimate the global production of “fake BC”. Biomass oxidation, as mimicked by our experimental systems, occurs as part of numerous biogeochemical processes (soil humification, aquatic photochemistry, microbial degradation, or mineral–organic matter interactions) with and without the presence of Fe, making this process relevant and extrapolatable to global scales.

#### For Soil Systems

By combining the annual rates of global aboveground litterfall (59 Pg-C)^62^ and belowground root-derived carbon production (25 Pg-C),^63^ we estimate that 84 Pg-C of fresh biomass are annually supplied to soils. As Fe-oxidized Pine 1 and Pine 2 had gained 2.44 and 2.33% ConAC over 12 years each, we can compute an average biomass-to-ConAC conversion rate of 0.195%/year (Table S1). By multiplying the biomass input to soils (84 Pg-C/year) with 0.195 ConAC%/year it can be estimated that 163 Tg-C of ConAC are annually produced from the oxidation of biomass in soils.

Another way to estimate global non-pyrogenic ConAC production is to use the experimental data from the sustained oxidation of maple wood (Figure 4A). The stable baseline value of 0.063% can be viewed as a quantitative estimate of the conversion of biomass to oxidation-resistant ConAC as it was produced during the first 6 h of the reaction and then persisted through the 10-day oxidation. The maximum biomass-to-ConAC conversion (0.217% at ∼18 h) can be an estimate of how much biomass can be converted to ConAC in a highly dynamic and radical-rich system (e.g., photochemically irradiated waters or mineral-rich soils). By accounting for the biomass conversion to oxidation-resistant ConAC (0.063%), we can estimate that the biomass conversion to oxidation-labile ConAC is 0.154 ± 0.008%. By multiplying the biomass input to soils (84 Pg-C/year) with the estimated non-pyrogenic ConAC production rates of oxidation-labile and oxidation-resistant ConAC (0.154 and 0.063%, respectively), we estimate that 129 Tg-C of oxidation-labile and 53 Tg-C of oxidation-resistant ConAC, totaling 182 Tg-C of ConAC, are annually produced from biomass in soils. For reference, pyrogenic inputs (i.e., BC) to soils are estimated to be 128 Tg-C/year,^13,14^ which is outweighed by our estimated non-pyrogenic ConAC input of 163–182 Tg-C/year. Thus, even though it is assumed that the ConAC in soils is *exclusively* derived from combustion (i.e., ConAC = BC),^8^ our results here show that it is possible that ConAC in soils could be entirely non-pyrogenic, except in areas with previous wildfire exposure.

#### For Fluvial Systems

The annual amount of DOC leached from terrestrial systems to waters is estimated to be 2.90 Pg-C.^64^ About 10% of this DOC leaching from soils is dConAC,^65^ i.e., 0.29 Pg-C. Assuming most soil-derived ConAC is non-pyrogenic, the annual seepage flux of non-pyrogenic ConAC into rivers would be 290 Tg-C/year. In addition, ConAC could be produced *in situ* in rivers from aquatic oxidation processes occurring photochemically, microbiologically, or through other abiotic processes, such as Fenton reactions on the surfaces of hydrologically mobilized minerals. The annual non-condensed biomass-derived DOC leaching from soils into waters is estimated to be 2.61 Pg (2.90 Pg total DOC – 0.29 Pg ConAC). By multiplying the amount of non-condensed DOC with the estimated biomass-to-ConAC conversion (0.195%/year), we estimate that 5.08 Tg-C of non-pyrogenic dConAC are annually produced in rivers. By using the ConAC conversion estimates from the sustained maple oxidation we can multiply the annual non-condensed biomass-DOC leaching into waters (2.61 Pg-C) with the estimated non-pyrogenic biomass conversion to oxidation-labile and oxidation-resistant dConAC (0.154 and 0.063%, respectively) to estimate that 4.01 Tg-C of oxidation-labile and 1.65 Tg-C of oxidation-resistant dConAC are annually produced in rivers. Thus, 5.66 Tg-C of the observed annual fluvial flux of dConAC can be river-sourced (produced *in situ*), which is comparable to the second estimate made above (5.08 Tg-C/year). For reference, the annual pyrogenic inputs (i.e., BC) to rivers are estimated to be 18 Tg-C.^13,66,67^ This number is relatively small, because ConAC experiences significant degradation during its fluvial transport. However, as the seepage flux from soils of non-pyrogenic ConAC is likely massive (290 Tg-C/year), and 5.08–5.66 Tg-C could be produced annually within rivers, even downstream, it is very likely that the majority of riverine ConAC is non-pyrogenic, except in areas where it is known that hydrology mobilizes pyrogenic products such as biochar from farms or lands that have recently experienced wildfires.

We recognize that soil and aquatic biogeochemical processes are complex, and the above calculations simplify them greatly. Because the non-pyrogenic formation of ConAC is highly understudied, many details about this process are unknown at present. This is why more complex modeling was not employed using the kinetic data from Figure 3 as many assumptions would have had to be made about environmental oxidation kinetics. The sustained Fenton oxidation (Figure 4A) was more representative, as hydroxyl radical fluxes are at steady-state concentrations in the environment. Notably, our estimates were based on Fenton oxidation of only two woody biomass types, which do not reflect the diversity of biomass materials found in the environment. However, these biomass types contain the most relevant environmental biopolymers (carbohydrates and lignin), making them useful primer models for estimating non-pyrogenic ConAC production as a first attempt. The non-pyrogenic ConAC estimates using the 12-year Fe-oxidized pine woods (163 Tg-C/year for soils, 5.08 Tg-C/year for rivers) compare very well to the non-pyrogenic ConAC estimates from the laboratory oxidation of maple wood (182 Tg-C/year for soils, 5.66 Tg-C/year for rivers). This validates our experimental approach and back-of-the-envelope approximations even though these results are in discrepancy with previously published estimates by Chen^30^ and Glaser,^68^ who argue that only up to 25% of soil ConAC inputs are non-pyrogenic. Unfortunately, at present, no methodology exists to distinguish pyrogenic from non-pyrogenic ConAC and thus, laboratory experiments and model systems, like our nail-wood systems, must be employed for studying this process at the risk of under/overestimates. The discrepancy between our and previously published works may be due to various reasons, including experimental designs (e.g., abiotic vs. biotic chemistries) or the use of different ConAC quantification methods (BPCA markers, BPCA-specific isotopes, NMR spectroscopy).

### Non-Pyrogenic ConAC Is Likely Prevalent in Terrestrial and Aquatic Systems

Much of the wildfire biogeochemistry research over the last 20 years has been based on the assumption that measured ConAC is *exclusively* derived from combustion processes and is equivalent to BC. Our findings challenge this assumption and show that plant litter and other biomass can be transformed into ConAC via oxidation with ROS. While we show this in model wood-nail systems as well as via experimental Fenton oxidations, ROS are naturally ubiquitous in terrestrial and aquatic environments with or without the presence of Fe. Thus, we expect non-pyrogenic production of ConAC to occur ubiquitously throughout the environment.

**Figure 5:**
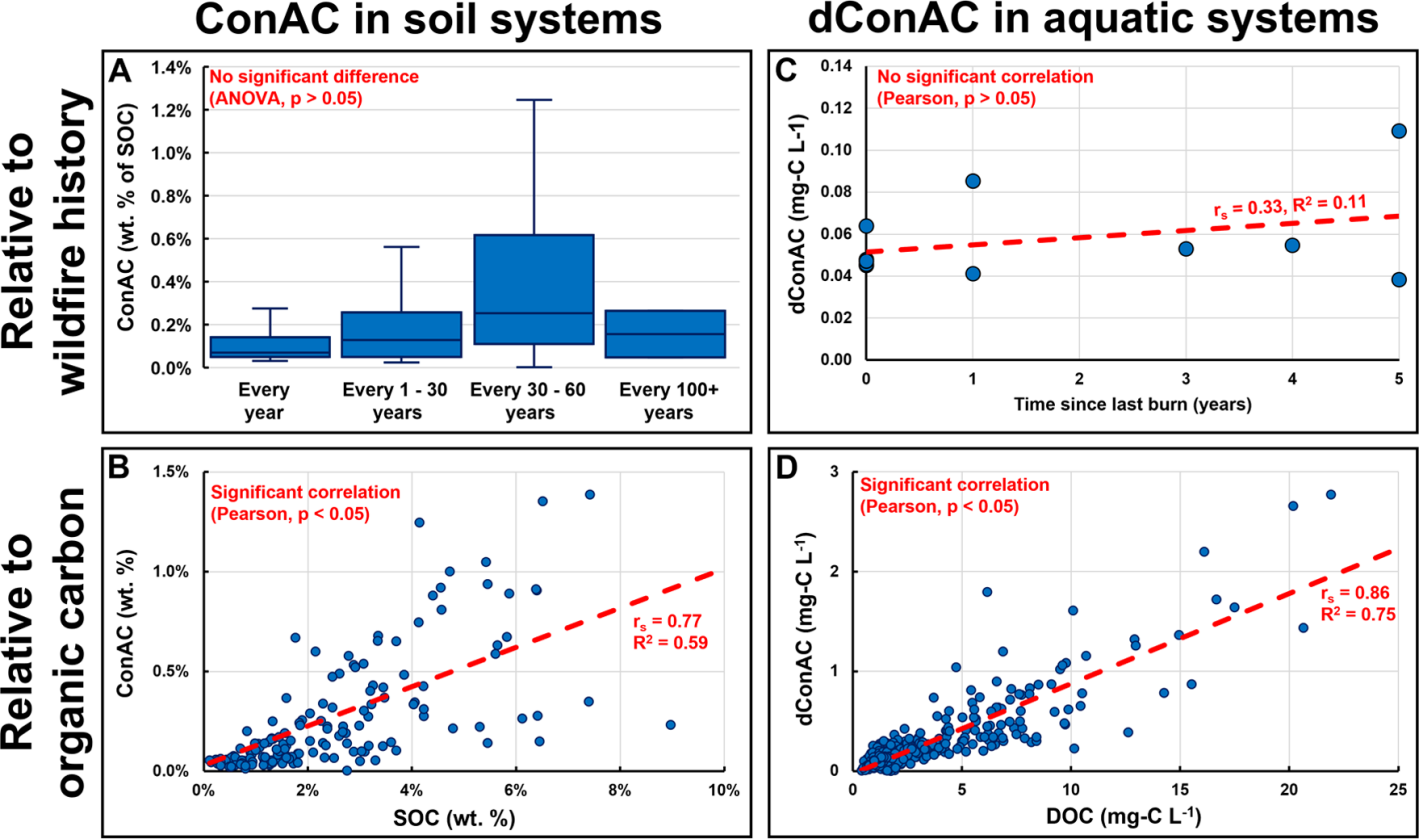
Previously published data showing that ConAC in soils (A) (data from Reisser^8^) or dConAC in aquatic systems (C) (data from Ding^16^) vary irrespectively of wildfire occurrence. (B, D) Coupling of ConAC and SOC quantities in soils (data from Reisser^8^) and dConAC and DOC quantities in aquatic systems (data from Jones^13^). Statistical analysis was done using the Toolbox for Environmental Research (TEnvR) in MATLAB.^79^

This proposition explains major discrepancies in the wildfire biogeochemistry literature. Reisser^8^ compiled 560 ConAC measurements in soils from 55 previously published studies revealing that ConAC did not covary with fire frequency (Figure 5A). Kane and Hockaday^15^ also evaluated several forest soils, which had been affected differently by fire events, and found no correlation between ConAC and wildfire history. One would expect that if ConAC in soils was *exclusively* pyrogenic, ConAC would strongly correlate with wildfire activity (i.e., more wildfires would have led to more production of ConAC). The lack of correlation is consistent with our proposition that ConAC in soil is primarily derived from a non-pyrogenic process, which has been also shown empirically recently.^31^ Furthermore, ConAC is strongly correlated with SOC regionally^18 −21^ and globally^8,22^ (Figure 5B). The current interpretation for this ConAC-SOC correlation is that charcoal from ancient fires (referred to as legacy BC) is equally distributed globally in SOC pools. However, the occurrence of wildfires is not distributed equally throughout the planet^14^ to result in globally equal proportions of BC to SOC. The atmospheric deposition flux of BC is also too limited (2–12 Tg-C/year)^61^ to cause an equally distributed BC input to soils. In light of our work presented here, it is much more likely that the ConAC-SOC correlations are due to the conversion of SOC to ConAC via oxidation occurring during the natural biogeochemical processing of organic matter in soils.

Similar inconsistencies can be found in the literature on dConAC in aquatic systems. Ding^16^ and Barton^17^ quantified dConAC in waters from watersheds that were subject to different fire frequencies and extents, respectively. It was found that fire history did not affect the concentrations of dConAC (Figure 5C)^16^ nor did a watershed burn extent gradient of 20–98%.^17^ One would have expected that with higher occurrences or extents of wildfires, more ConAC would be produced and solubilized as dConAC. However, this was not observed by Ding^16^ nor Barton^17^ suggesting that the dynamics of dConAC are not linked with wildfires and that dConAC varies independently of fire events. Another odd observation was that streams in a forested watershed in Japan contained dConAC of up to 6% of DOC with no wildfire activities for at least 110 years.^25^ Like regional^18−21^ and global^8,22^ ConAC-SOC correlations, dConAC and DOC are also strongly correlated in regional^16,69−72^ and global^13,23,73^ aquatic environments (Figure 5D). Currently, this dConAC-DOC correlation is interpreted as “equally distributed” BC in soils leached by rainwater and groundwater at constant rates to be entrained in natural waters. This still does not agree with the argument that BC is *not* equally distributed in terrestrial environments,^14^ because wildfires do not occur homogeneously on the planet.^74^ Furthermore, this interpretation assumes that charcoal (BC) can constantly leach dissolved BC. Laboratory studies of charcoal leaching reveal that charcoal leaches very little dissolved BC, and in fact, that leaching fluxes are not continuous over long time scales.^55^ This dConAC-DOC correlation can be alternatively explained by our proposition that (1) SOC is converted to ConAC, and both leach from soils to rivers causing their dissolved fluvial concentrations to covary, and that (2) DOC is converted to dConAC via *in situ* oxidative pathways.

The observation of oxidation-resistant ConAC (Figure 4A) can explain the accumulation of ConAC in terrestrial and marine environments. At present, ConAC removal fluxes exceed ConAC input fluxes for oceanic systems,^61^ indicating that there is a source of dConAC that has not been reconciled. Biomass oxidation is likely the missing piece of this mass balance (e.g., conversion of algal biomass to dConAC in surface waters). Furthermore, the deep ocean contains large amounts of dConAC (∼14 Pg-C) of ancient radiocarbon age (>20,000 ^14^C years).^75^ This persistence of dConAC is also enigmatic, as riverine fluxes are sufficient to sustain the turnover of the entire oceanic dConAC pool in just 500 years, suggesting that the radiocarbon age of oceanic dConAC should be young. As ConAC is biorefractory^76,77^ (i.e., resistant to biodegradation), and per our findings, some fraction of it is also chemically refractory (i.e., resistant to oxidation), a ConAC fraction may survive the redox gradients in environmental systems, allowing ConAC and its dConAC fraction to accumulate and remain extremely stable in soils, sediments, and the deep ocean for millennia. This agrees with the radiocarbon data for ConAC in these environments.^78^

Collectively, our findings explain better the ConAC-SOC and dConAC-DOC correlations as well as the global accumulation of ConAC. Thus, the current views in wildfire biogeochemistry need to be amended to consider the presented novel perspective of non-pyrogenic ConAC formation and persistence during oxidation. This should be accounted for in the nomenclature, analytical methods, and future research trajectories pertaining to wildfire biogeochemistry to properly constrain the role of wildfires in the environment, as well as to accurately synthesize global biogeochemical cycle models of carbon or its compound classes such as ConAC.

### Black Carbon (BC) Has Become a Deceiving Terminology Based on Overextrapolated Structure–Function Relationship

Since its first definition by Goldberg,^1^ BC has been widely used synonymously with pyrogenic carbon (pyC) to describe organic material formed by wildfires, fossil fuels combustion, or anthropogenic production of biochar. It is highly appropriate that the products from these pyrolytic processes are labeled as BC or pyC. Since charcoals are primarily composed of ConAC, it has been assumed that ConAC found in terrestrial or aquatic environments must be also of pyrogenic origin. Thus, a structure-relationship of ConAC = BC (or ConAC = pyC) has been developed over the years and employed globally. The quantitative results in our study show that ConAC may be largely of non-pyrogenic origin as previously suggested by qualitative studies.^30,31,56^ The BC or pyC terminologies would have been inappropriate for our study as they would have imposed a pyrogenic source onto non-pyrogenically produced ConAC. It has been also shown that ConAC is present in petroleum,^80^ asphalt,^81^ and hydrothermal vent exhaust,^81^ which exemplifies how, if the BC or pyC terminologies were used for ConAC in such samples, they would imposed a pyrogenic source to petrogenic molecules.

Another common misconception is that the methods employed by wildfire researchers (BPCA, chemothermal oxidation, and others) detect pyrogenic molecules (i.e., BC) in environmental matrices. There is an expanding literature raising awareness that “BC methods” in fact do not measure fire-derived residues, but detect a certain type of structures (ConAC) in the analyzed sample.^22,82,83^ In light of our findings, assuming that BPCA (and other) methods quantify BC would lead to major overestimations of environmental reservoirs and fluxes of “true” fire-derived carbon.^56^ This is especially notable for soils, where the non-pyrogenic process of soil formation (soil humification) produces ConAC,^31^ with biomass oxidation being a key process. Thus, it is very likely that the majority of reported BC quantities in soil systems (Figure 5A,B), in fact, correspond to refractory humic ConAC and not BC. In summary, the structure–function relationship between BPCA measurements (ConAC) and pyrolysis is overextrapolated, and many previous studies using BC and pyC terminologies deceptively impose a pyrogenic source onto ConAC measurements, which source assignment may or may not be true for the particular samples or environmental system.

To resolve this issue, we recommend the use of terminology that is specifically tied to the analytical method. We can learn lessons from the community using fluorescence spectroscopy to study dissolved organic matter. It was assumed at first that peak M in fluorescence spectra corresponded to exudates of marine microbes. This structure–function relationship was later found to be faulty when peak M was reported in various terrestrial and freshwater environments. Thus, for measurements of the BPCA method, we recommend the use of ConAC, for which the nomenclature remains neutral regarding source. It will be up to the researcher to determine if ConAC is equivalent to BC depending on their study. We believe that terminologies such as BC and pyC should be used only in cases when there is significant confidence that the measured ConAC is fire-derived. Compound-specific isotopic measurements (e.g., ConAC-δ^13^C)^53,80^ may be able to assist in differentiating pyrogenic, petrogenic, and non-pyrogenic sources of ConAC though this remains to be tested. Overall, the interpretation of ConAC measurements from BPCA or other methods should be performed carefully, and non-pyrogenic sources must be considered in order to properly link ConAC dynamics with wildfires or other pyrolytic/thermogenic activities and constrain this refractory group of molecules within the global carbon cycle.

### Emerging Directions for Future Research

Considering that we show potentially massive non-pyrogenic ConAC fluxes into soils and fluvial environments, it is important that future studies consider non-pyrogenic contributions interfering with wildfire-derived carbon measurements. Future studies should establish robust non-pyrogenic ConAC production rates from various biomass, by other ROS (singlet oxygen, superoxide, etc.),^84^ as well as perform long-term oxidation experiments of SOC suspensions and DOC solutions under different conditions (photochemical, microbial). Instead of quantifying total ConAC, as we have done here, future studies should also consider leaching experiments to determine the production rates of dConAC relative to the particulate ConAC fraction. Lastly, future studies should also aim to develop novel methods^85,86^ that can accurately identify wildfire-derived species (i.e., true BC) that cannot be produced from other environmental processes. Conversely, methodologies for identifying and quantifying non-pyrogenic ConAC are also critically needed to properly differentiate pyrogenic from non-pyrogenic ConAC and accurately constrain the impact of wildfires on the global environment.

## Data Availability

Molecular formula catalogs and raw BPCA data have been published in the Mendeley Data Repository (doi: 10.17632/9mj4dvmbt5.2). Any other ancillary measurements used in this publication were provided in the manuscript or its Supporting Information. Parts of this work were also previously published as part of Aleksandar I. Goranov’s dissertation (doi: 10.25777/fpsv-4e28)^45^ and are reused with the appropriate permissions.

## Supporting Information Available

Pictures of additional wood specimens; materials and methods for supplementary analyses; bulk elemental characteristics of wood specimens; supplementary NMR, mass spectrometric (molecular and metals) and X-ray Fe and Cu distribution and speciation results and discussions; effect of pressure treatment on ConAC formation chemistry; and raw data from laboratory Fenton experiments (PDF)

## References

[ref1] Goldberg E. D. Black Carbon in the Environment: Properties and Distribution; J. Wiley, 1985.

[ref2] Hedges J. I.; Eglinton G.; Hatcher P. G.; Kirchman D. L.; Arnosti C.; Derenne S.; Evershed R. P.; Kogel-Knabner I.; de Leeuw J. W.; Littke R.; Michaelis W.; Rullkotter J. The molecularly-uncharacterized component of nonliving organic matter in natural environments. Org. Geochem. 2000, 31 (10), 945–958. 10.1016/S0146-6380(00)00096-6.

[ref3] Baldock J. A.; Smernik R. J. Chemical composition and bioavailability of thermally, altered *Pinus resinosa* (Red Pine) wood. Org. Geochem. 2002, 33 (9), 1093–1109. 10.1016/S0146-6380(02)00062-1.

[ref4] Santín C.; Doerr S. H.; Merino A.; Bryant R.; Loader N. J. Forest floor chemical transformations in a boreal forest fire and their correlations with temperature and heating duration. Geoderma 2016, 264, 71–80. 10.1016/j.geoderma.2015.09.021.

[ref5] Masiello C. A. New directions in black carbon organic geochemistry. Mar. Chem. 2004, 92 (1–4), 201–213. 10.1016/j.marchem.2004.06.043.

[ref6] Czimczik C. I.; Masiello C. A. Controls on black carbon storage in soils. Global Biogeochem. Cycles 2007, 21 (3), GB3005 10.1029/2006GB002798.

[ref7] Bird M. I.; Wynn J. G.; Saiz G.; Wurster C. M.; McBeath A. The pyrogenic carbon cycle. Annu. Rev. Earth Planet. Sci. 2015, 43 (1), 273–298. 10.1146/annurev-earth-060614-105038.

[ref8] Reisser M.; Purves R. S.; Schmidt M. W. I.; Abiven S. Pyrogenic carbon in soils: A literature-based inventory and a global estimation of its content in soil organic carbon and stocks. Front. Earth Sci. 2016, 4, 80 10.3389/feart.2016.00080.

[ref9] Santín C.; Doerr S. H.; Kane E. S.; Masiello C. A.; Ohlson M.; de la Rosa J. M.; Preston C. M.; Dittmar T. Towards a global assessment of pyrogenic carbon from vegetation fires. Global Change Biol. 2016, 22 (1), 76–91. 10.1111/gcb.12985. 26010729

[ref10] Wozniak A. S.; Bauer J. E.; Sleighter R. L.; Dickhut R. M.; Hatcher P. G. Technical Note: Molecular characterization of aerosol-derived water soluble organic carbon using ultrahigh resolution electrospray ionization Fourier transform ion cyclotron resonance mass spectrometry. Atmos. Chem. Phys. 2008, 8 (17), 5099–5111. 10.5194/acp-8-5099-2008.

[ref11] Bao H.; Niggemann J.; Luo L.; Dittmar T.; Kao S. J. Aerosols as a source of dissolved black carbon to the ocean. Nat. Commun. 2017, 8 (1), 510 10.1038/s41467-017-00437-3. 28894096 PMC5593878

[ref12] Wagner S.; Jaffe R.; Stubbins A. Dissolved black carbon in aquatic ecosystems. Limnol. Oceanogr. Lett. 2018, 3 (3), 168–185. 10.1002/lol2.10076.

[ref13] Jones M. W.; Coppola A. I.; Santin C.; Dittmar T.; Jaffe R.; Doerr S. H.; Quine T. A. Fires prime terrestrial organic carbon for riverine export to the global oceans. Nat. Commun. 2020, 11 (1), 2791 10.1038/s41467-020-16576-z. 32494057 PMC7270114

[ref14] Jones M. W.; Santin C.; van der Werf G. R.; Doerr S. H. Global fire emissions buffered by the production of pyrogenic carbon. Nat. Geosci. 2019, 12 (9), 742–747. 10.1038/s41561-019-0403-x.

[ref15] Kane E. S.; Hockaday W. C.; Turetsky M. R.; Masiello C. A.; Valentine D. W.; Finney B. P.; Baldock J. A. Topographic controls on black carbon accumulation in Alaskan black spruce forest soils: implications for organic matter dynamics. Biogeochemistry 2010, 100 (1–3), 39–56. 10.1007/s10533-009-9403-z.

[ref16] Ding Y.; Yamashita Y.; Dodds W. K.; Jaffe R. Dissolved black carbon in grassland streams: Is there an effect of recent fire history?. Chemosphere 2013, 90 (10), 2557–2562. 10.1016/j.chemosphere.2012.10.098. 23219080

[ref17] Barton R.; Richardson C. M.; Pae E.; Montalvo M. S.; Redmond M.; Zimmer M. A.; Wagner S. Hydrology, rather than wildfire burn extent, determines post-fire organic and black carbon export from mountain rivers in central coastal California. Limnol. Oceanogr. Lett. 2024, 9, 70–80. 10.1002/lol2.10360.

[ref18] Glaser B.; Amelung W. Pyrogenic carbon in native grassland soils along a climosequence in North America. Global Biogeochem. Cycles 2003, 17 (2), 1064 10.1029/2002GB002019.

[ref19] Jauss V.; Johnson M.; Krull E.; Daub M.; Lehmann J. Pyrogenic carbon controls across a soil catena in the Pacific Northwest. Catena 2015, 124, 53–59. 10.1016/j.catena.2014.09.001.

[ref20] Ahmed Z. U.; Woodbury P. B.; Sanderman J.; Hawke B.; Jauss V.; Solomon D.; Lehmann J. Assessing soil carbon vulnerability in the Western USA by geospatial modeling of pyrogenic and particulate carbon stocks. J. Geophys. Res.: Biogeosci. 2017, 122 (2), 354–369. 10.1002/2016JG003488.

[ref21] Qi F.; Naidu R.; Bolan N. S.; Dong Z.; Yan Y.; Lamb D.; Bucheli T. D.; Choppala G.; Duan L.; Semple K. T. Pyrogenic carbon in Australian soils. Sci. Total Environ. 2017, 586, 849–857. 10.1016/j.scitotenv.2017.02.064. 28215804

[ref22] Zimmerman A. R.; Mitra S. Trial by fire: On the terminology and methods used in pyrogenic organic carbon research. Front. Earth Sci. 2017, 5, 95 10.3389/feart.2017.00095.

[ref23] Jaffé R.; Ding Y.; Niggemann J.; Vahatalo A. V.; Stubbins A.; Spencer R. G.; Campbell J.; Dittmar T. Global charcoal mobilization from soils via dissolution and riverine transport to the oceans. Science 2013, 340 (6130), 345–347. 10.1126/science.1231476. 23599492

[ref24] Tadini A. M.; Martin-Neto L.; Goranov A. I.; Milori D. M. B. P.; Bernardi A. C. C.; Oliveira P. P. A.; Pezzopane J. R. M.; Colnago L. A.; Hatcher P. G. Chemical characteristics of soil organic matter from integrated agricultural systems in southeastern Brazil. Eur. J. Soil Sci. 2022, 73 (1), 13136 10.1111/ejss.13136.

[ref25] Yamashita Y.; Kojima D.; Yoshida N.; Shibata H. Relationships between dissolved black carbon and dissolved organic matter in streams. Chemosphere 2021, 271, 129824 10.1016/j.chemosphere.2021.129824. 33736211

[ref26] Chen H. M.; Abdulla H. A. N.; Sanders R. L.; Myneni S. C. B.; Mopper K.; Hatcher P. G. Production of black carbon-like and aliphatic molecules from terrestrial dissolved organic matter in the presence of sunlight and iron. Environ. Sci. Technol. Lett. 2014, 1 (10), 399–404. 10.1021/ez5002598.

[ref27] Waggoner D. C.; Chen H. M.; Willoughby A. S.; Hatcher P. G. Formation of black carbon-like and alicyclic aliphatic compounds by hydroxyl radical initiated degradation of lignin. Org. Geochem. 2015, 82, 69–76. 10.1016/j.orggeochem.2015.02.007.

[ref28] Fenton H. J. H. LXXIII. - Oxidation of tartaric acid in presence of iron. J. Chem. Soc., Trans. 1894, 65 (0), 899–910. 10.1039/CT8946500899.

[ref29] Walling C. Fenton’s reagent revisited. Acc. Chem. Res. 1975, 8 (4), 125–131. 10.1021/ar50088a003.

[ref30] Chen X.; Ye X.; Chu W.; Olk D. C.; Cao X.; Schmidt-Rohr K.; Zhang L.; Thompson M. L.; Mao J.; Gao H. Formation of char-like, fused-ring aromatic structures from a nonpyrogenic pathway during decomposition of wheat straw. J. Agric. Food Chem. 2020, 68 (9), 2607–2614. 10.1021/acs.jafc.9b06037. 32096642

[ref31] Gamage J.; Voroney P.; Gillespie A.; Lo A.; Longstaffe J. Evidence for the formation of fused aromatic ring structures in an organic soil profile in the early diagenesis. Sci. Rep. 2023, 13 (1), 12378 10.1038/s41598-023-39181-8. 37524728 PMC10390584

[ref32] Bodek I.; Lyman W. J.; Reehl W. F.; Rosenblatt D. H. Environmental Inorganic Chemistry: Properties, Processes, And Estimation Methods; Pergamon Press, 1988. 10.1016/c2009-0-07927-6.

[ref33] Yu G. H.; Kuzyakov Y. Fenton chemistry and reactive oxygen species in soil: Abiotic mechanisms of biotic processes, controls and consequences for carbon and nutrient cycling. Earth-Sci. Rev. 2021, 214, 103525 10.1016/j.earscirev.2021.103525.

[ref34] Scully N. M.; Cooper W. J.; Tranvik L. J. Photochemical effects on microbial activity in natural waters: The interaction of reactive oxygen species and dissolved organic matter. FEMS Microbiol. Ecol. 2003, 46 (3), 353–357. 10.1016/S0168-6496(03)00198-3. 19719565

[ref35] McNally A. M.; Moody E. C.; McNeill K. Kinetics and mechanism of the sensitized photodegradation of lignin model compounds. Photochem. Photobiol. Sci. 2005, 4 (3), 268–274. 10.1039/b416956e. 15738994

[ref36] Porcal P.; Dillon P. J.; Molot L. A. Photochemical production and decomposition of particulate organic carbon in a freshwater stream. Aquat. Sci. 2013, 75 (4), 469–482. 10.1007/s00027-013-0293-8.

[ref37] Xiao Y.; Carena L.; Nasi M. T.; Vahatalo A. V. Superoxide-driven autocatalytic dark production of hydroxyl radicals in the presence of complexes of natural dissolved organic matter and iron. Water Res. 2020, 177, 115782 10.1016/j.watres.2020.115782. 32294593

[ref38] Trusiak A.; Treibergs L.; Kling G.; Cory R. The controls of iron and oxygen on hydroxyl radical (•OH) production in soils. Soil Syst. 2019, 3, 1 10.3390/soilsystems3010001.

[ref39] Billen G.; Servais P.; Becquevort S. Dynamics of bacterioplankton in oligotrophic and eutrophic aquatic environments: Bottom-up or top-down control?. Hydrobiologia 1990, 207 (1), 37–42. 10.1007/BF00041438.

[ref40] Higuchi T. Microbial degradation of lignin: Role of lignin peroxidase, manganese peroxidase, and laccase. Proc. Jpn. Acad., Ser. B 2004, 80 (5), 204–214. 10.2183/pjab.80.204.

[ref41] Hyde S. M.; Wood P. M. A mechanism for production of hydroxyl radicals by the brown-rot fungus *Coniophora Puteana*: Fe(III) reduction by cellobiose dehydrogenase and Fe(II) oxidation at a distance from the hyphae. Microbiology 1997, 143 (1), 259–266. 10.1099/00221287-143-1-259. 33711854

[ref42] Wang M.; Liu Y.; Shi H.; Li S.; Chen S. Yielding hydroxyl radicals in the Fenton-like reaction induced by manganese (II) oxidation determines Cd mobilization upon soil aeration in paddy soil systems. Environ. Pollut. 2022, 292, 118311 10.1016/j.envpol.2021.118311. 34627964

[ref43] Stohs S. J.; Bagchi D. Oxidative mechanisms in the toxicity of metal ions. Free Radical Biol. Med. 1995, 18 (2), 321–336. 10.1016/0891-5849(94)00159-H. 7744317

[ref44] Haber F.; Weiss J. Über die katalyse des hydroperoxydes. Naturwissenschaften 1932, 20 (51), 948–950. 10.1007/BF01504715.

[ref45] Goranov A. I. Advances in the understanding of sourcing and fate of pyrogenic organic matter in the environment, Doctoral Dissertation; Old Dominion University: Norfolk, VA, 2020 10.25777/fpsv-4e28.

[ref46] Campbell H.; Nizamani R.; Jones S. W.; Williams F. N. Death due to fractal wood burning: An emerging public health problem. Journal of Burn Care & Research 2020, 41 (4), 788–790. 10.1093/jbcr/iraa066. 32353877

[ref47] Obeid W.; Salmon E.; Hatcher P. G. The effect of different isolation procedures on algaenan molecular structure in Scenedesmus green algae. Org. Geochem. 2014, 76, 259–269. 10.1016/j.orggeochem.2014.09.004.

[ref48] Hartman B. E. Artificial maturation studies of polymethylenic plant biopolymers: Investigating the chemical alterations from plant material to coal. Doctoral Dissertation, Old Dominion University, Norfolk, VA, 2015.

[ref49] Serra O.; Geldner N. The making of suberin. New Phytol. 2022, 235 (3), 848–866. 10.1111/nph.18202. 35510799 PMC9994434

[ref50] Khatami S.; Deng Y.; Tien M.; Hatcher P. G. Formation of water-soluble organic matter through fungal degradation of lignin. Org. Geochem. 2019, 135, 64–70. 10.1016/j.orggeochem.2019.06.004.

[ref51] Koenig A. B.; Sleighter R. L.; Salmon E.; Hatcher P. G. NMR structural characterization of *Quercus alba* (White Oak) degraded by the brown rot fungus, *Laetiporus sulphureus*. Journal of Wood Chemistry and Technology 2010, 30 (1), 61–85. 10.1080/02773810903276668.

[ref52] Kappenberg A.; Blasing M.; Lehndorff E.; Amelung W. Black carbon assessment using benzene polycarboxylic acids: Limitations for organic-rich matrices. Org. Geochem. 2016, 94, 47–51. 10.1016/j.orggeochem.2016.01.009.

[ref53] Wagner S.; Brandes J.; Goranov A. I.; Drake T. W.; Spencer R. G. M.; Stubbins A. Online quantification and compound-specific stable isotopic analysis of black carbon in environmental matrices via liquid chromatography-isotope ratio mass spectrometry. Limnol. Oceanogr.: Methods 2017, 15 (12), 995–1006. 10.1002/lom3.10219.

[ref54] Stubbins A.; Niggemann J.; Dittmar T. Photo-lability of deep ocean dissolved black carbon. Biogeosciences 2012, 9 (5), 1661–1670. 10.5194/bg-9-1661-2012.

[ref55] Bostick K. W.; Zimmerman A. R.; Wozniak A. S.; Mitra S.; Hatcher P. G. Production and composition of pyrogenic dissolved organic matter from a logical series of laboratory-generated chars. Front. Earth Sci. 2018, 6, 43 10.3389/feart.2018.00043.

[ref56] Gerke J. Black (pyrogenic) carbon in soils and waters: A fragile data basis extensively interpreted. Chem. Biol. Technol. Agric. 2019, 6, 13 10.1186/s40538-019-0151-6.

[ref57] Tadini A. M.; Goranov A. I.; Martin-Neto L.; Bernardi A. C. C.; Oliveira P. P. A.; Pezzopane J. R. M.; Hatcher P. G. Structural characterization using 2D NMR spectroscopy and TMAH-GC x GC-MS: Application to humic acids from soils of an integrated agricultural system and an Atlantic native forest. Sci. Total Environ. 2022, 815, 152605 10.1016/j.scitotenv.2021.152605. 34971684

[ref58] Bostick K. W.; Zimmerman A. R.; Goranov A. I.; Mitra S.; Hatcher P. G.; Wozniak A. S. Photolability of pyrogenic dissolved organic matter from a thermal series of laboratory-prepared chars. Sci. Total Environ. 2020, 724, 1–9. 10.1016/j.scitotenv.2020.138198. 32272404

[ref59] Goranov A. I.; Wozniak A. S.; Bostick K. W.; Zimmerman A. R.; Mitra S.; Hatcher P. G. Photochemistry after fire: Structural transformations of pyrogenic dissolved organic matter elucidated by advanced analytical techniques. Geochim. Cosmochim. Acta 2020, 290, 271–292. 10.1016/j.gca.2020.08.030.

[ref60] Mannino A.; Harvey H. R. Black carbon in estuarine and coastal ocean dissolved organic matter. Limnol. Oceanogr. 2004, 49 (3), 735–740. 10.4319/lo.2004.49.3.0735.

[ref61] Coppola A. I.; Wagner S.; Lennartz S. T.; Seidel M.; Ward N. D.; Dittmar T.; Santin C.; Jones M. W. The black carbon cycle and its role in the Earth system. Nat. Rev. Earth Eniron. 2022, 3 (8), 516–532. 10.1038/s43017-022-00316-6.

[ref62] Houghton R. A. Balancing the global carbon budget. Annu. Rev. Earth Planet. Sci. 2007, 35 (1), 313–347. 10.1146/annurev.earth.35.031306.140057.

[ref63] Gherardi L. A.; Sala O. E. Global patterns and climatic controls of belowground net carbon fixation. Proc. Natl. Acad. Sci. U.S.A. 2020, 117 (33), 20038–20043. 10.1073/pnas.2006715117. 32747527 PMC7443884

[ref64] Tranvik L. J.; Downing J. A.; Cotner J. B.; Loiselle S. A.; Striegl R. G.; Ballatore T. J.; Dillon P.; Finlay K.; Fortino K.; Knoll L. B.; Kortelainen P. L.; Kutser T.; Larsen S.; Laurion I.; Leech D. M.; McCallister S. L.; McKnight D. M.; Melack J. M.; Overholt E.; Porter J. A.; Prairie Y.; Renwick W. H.; Roland F.; Sherman B. S.; Schindler D. W.; Sobek S.; Tremblay A.; Vanni M. J.; Verschoor A. M.; von Wachenfeldt E.; Weyhenmeyer G. A. Lakes and reservoirs as regulators of carbon cycling and climate. Limnol. Oceanogr. 2009, 54 (6), 2298–2314. 10.4319/lo.2009.54.6_part_2.2298.

[ref65] Wagner S.; Ding Y.; Jaffe R. A new perspective on the apparent solubility of dissolved black carbon. Front. Earth Sci. 2017, 5, 75 10.3389/feart.2017.00075.

[ref66] Wang X. C.; Xu C. L.; Druffel E. M.; Xue Y. J.; Qi Y. Z. Two black carbon pools transported by the Changjiang and Huanghe Rivers in China. Global Biogeochem. Cycles 2016, 30 (12), 1778–1790. 10.1002/2016GB005509.

[ref67] Qi Y.; Fu W.; Tian J.; Luo C.; Shan S.; Sun S.; Ren P.; Zhang H.; Liu J.; Zhang X.; Wang X. Dissolved black carbon is not likely a significant refractory organic carbon pool in rivers and oceans. Nat. Commun. 2020, 11 (1), 5051 10.1038/s41467-020-18808-8. 33028806 PMC7541478

[ref68] Glaser B.; Knorr K. H. Isotopic evidence for condensed aromatics from non-pyrogenic sources in soils - implications for current methods for quantifying soil black carbon. Rapid Commun. Mass Spectrom. 2008, 22 (7), 935–942. 10.1002/rcm.3448. 18306211

[ref69] Dittmar T.; de Rezende C. E.; Manecki M.; Niggemann J.; Ovalle A. R. C.; Stubbins A.; Bernardes M. C. Continuous flux of dissolved black carbon from a vanished tropical forest biome. Nat. Geosci. 2012, 5 (9), 618–622. 10.1038/Ngeo1541.

[ref70] Ding Y.; Yamashita Y.; Jones J.; Jaffe R. Dissolved black carbon in boreal forest and glacial rivers of central Alaska: Assessment of biomass burning versus anthropogenic sources. Biogeochemistry 2015, 123 (1–2), 15–25. 10.1007/s10533-014-0050-7.

[ref71] Ding Y.; Cawley K. M.; da Cunha C. N.; Jaffe R. Environmental dynamics of dissolved black carbon in wetlands. Biogeochemistry 2014, 119 (1–3), 259–273. 10.1007/s10533-014-9964-3.

[ref72] Güereña D. T.; Lehmann J.; Walter T.; Enders A.; Neufeldt H.; Odiwour H.; Biwott H.; Recha J.; Shepherd K.; Barrios E.; Wurster C. Terrestrial pyrogenic carbon export to fluvial ecosystems: Lessons learned from the White Nile watershed of East Africa. Global Biogeochem. Cycles 2015, 29 (11), 1911–1928. 10.1002/2015GB005095.

[ref73] Wagner S.; Riedel T.; Niggemann J.; Vahatalo A. V.; Dittmar T.; Jaffe R. Linking the molecular signature of heteroatomic dissolved organic matter to watershed characteristics in world rivers. Environ. Sci. Technol. 2015, 49 (23), 13798–13806. 10.1021/acs.est.5b00525. 26153846

[ref74] Bowring S. P. K.; Jones M.; Ciais P.; Guenet B.; Abiven S. Pyrogenic carbon decomposition critical to resolving fire’s role in the Earth system. Nat. Geosci. 2022, 15, 135–142. 10.1038/s41561-021-00892-0.

[ref75] Coppola A. I.; Druffel E. R. M. Cycling of black carbon in the ocean. Geophys. Res. Lett. 2016, 43 (9), 4477–4482. 10.1002/2016GL068574.

[ref76] Kuzyakov Y.; Bogomolova I.; Glaser B. Biochar stability in soil: Decomposition during eight years and transformation as assessed by compound-specific ^14^C analysis. Soil Biology and Biochemistry 2014, 70, 229–236. 10.1016/j.soilbio.2013.12.021.

[ref77] Bostick K. W.; Zimmerman A. R.; Goranov A. I.; Mitra S.; Hatcher P. G.; Wozniak A. S. Biolability of fresh and photodegraded pyrogenic dissolved organic matter from laboratory-prepared chars. J. Geophys. Res.: Biogeosci. 2021, 126 (5), e2020JG005981 10.1029/2020JG005981.

[ref78] Coppola A. I.; Wiedemeier D. B.; Galy V.; Haghipour N.; Hanke U. M.; Nascimento G. S.; Usman M.; Blattmann T. M.; Reisser M.; Freymond C. V.; Zhao M.; Voss B.; Wacker L.; Schefuß E.; Peucker-Ehrenbrink B.; Abiven S.; Schmidt M. W. I.; Eglinton T. I. Global-scale evidence for the refractory nature of riverine black carbon. Nat. Geosci. 2018, 11 (8), 584–588. 10.1038/s41561-018-0159-8.

[ref79] Goranov A. I.; Sleighter R. L.; Yordanov D. A.; Hatcher P. TEnvR: MATLAB-based toolbox for environmental research. Anal. Methods 2023, 15 (15), 5390–5400. 10.1039/D3AY00750B. 37807701

[ref80] Goranov A. I.; Schaller M. F.; Long J. A.; Podgorski D. C.; Wagner S. Characterization of asphaltenes and petroleum using benzenepolycarboxylic acids (BPCAs) and compound-specific stable carbon isotopes. Energy Fuels 2021, 35 (22), 18135–18145. 10.1021/acs.energyfuels.1c02374.

[ref81] Brünjes J.; Seidel M.; Dittmar T.; Niggemann J.; Schubotz F. Natural asphalt seeps are potential sources for recalcitrant oceanic dissolved organic sulfur and dissolved black carbon. Environ. Sci. Technol. 2022, 56 (12), 9092–9102. 10.1021/acs.est.2c01123. 35584055

[ref82] Chang Z.; Tian L.; Li F.; Zhou Y.; Wu M.; Steinberg C. E. W.; Dong X.; Pan B.; Xing B. Benzene polycarboxylic acid - A useful marker for condensed organic matter, but not for only pyrogenic black carbon. Sci. Total Environ. 2018, 626, 660–667. 10.1016/j.scitotenv.2018.01.145. 29898553

[ref83] Vaezzadeh V.; Zhong G.; Zhang G. Benzene polycarboxylic acids as molecular markers of black carbon: Progresses and challenges. Chemosphere 2023, 341, 140112 10.1016/j.chemosphere.2023.140112. 37689153

[ref84] Waggoner D. C.; Wozniak A. S.; Cory R. M.; Hatcher P. G. The role of reactive oxygen species in the degradation of lignin derived dissolved organic matter. Geochim. Cosmochim. Acta 2017, 208, 171–184. 10.1016/j.gca.2017.03.036.

[ref85] Thurman E. M.; Ferrer I.; Bowden M.; Mansfeldt C.; Fegel T. S.; Rhoades C. C.; Rosario-Ortiz F. Occurrence of benzene polycarboxylic acids in ash and streamwater after the Cameron Peak Fire. Environ. Sci. Technol. Water 2023, 3, 3848 10.1021/acsestwater.3c00246.

[ref86] Ferrer I.; Thurman E. M.; Zweigenbaum J. A.; Murphy S. F.; Webster J. P.; Rosario-Ortiz F. L. Wildfires: Identification of a new suite of aromatic polycarboxylic acids in ash and surface water. Sci. Total Environ. 2021, 770, 144661 10.1016/j.scitotenv.2020.144661. 33513501

