# Supplementary material for: Potentially Massive and Global Non-Pyrogenic Production of Condensed “Black” Carbon through Biomass Oxidation
Source: Environ Sci Technol. 2024 Jan 31;58(6):2750–61. doi: 10.1021/acs.est.3c05448 (PMC10867845; doi:10.1021/acs.est.3c05448)
Supplement: Supplementary file 1 — es3c05448_si_001.pdf [file es3c05448_si_001.pdf]

Potentially Massive and Global Non-Pyrogenic Production of Condensed "Black" Carbon through Biomass Oxidation

Aleksandar I. Goranov[a,*], Hongmei Chen[a,1], Jianshu Duan[b], Satish C. B. Myneni[b], Patrick G. Hatcher[a,*]

[a]Department of Chemistry and Biochemistry, Old Dominion University, Norfolk, VA 23529 USA
[b]Department of Geosciences, Princeton University, Princeton, NJ 08544 USA

Corresponding Authors (*):

- Aleksandar I. Goranov: 4501 Elkhorn Ave, CHEM 3000A, Norfolk, VA 23529, Email: aleksandar.i.goranov@gmail.com; Phone: (201) 647-8380; Fax: (757) 683 - 4628
- Patrick G. Hatcher: 4501 Elkhorn Ave, CHEM 3000B, Norfolk, VA 23529; Email: phatcher@odu.edu; Phone: (757) 683 – 6537; Fax: (757) 683 - 4628

Supporting Information (SI)

Section 1. Additional wood specimens (Pine 2 and Maple): **Figure S1** S2
Section 2. Materials and methods for supplementary analyses S3
2.1. Quantification of organic carbon and hydrogen S3
2.2. Quantification of metals S3
2.3. Solid-state nuclear magnetic resonance (NMR) analysis S4
2.4. X-ray fluorescence imaging and Fe X-ray absorption near edge structure (XANES) S4
2.5. Extraction of organic matter S4
2.6. Ultrahigh resolution mass spectrometry (FT-ICR-MS) S5
Section 3. Bulk elemental characteristics: **Table S1** S6
Section 4. Structural characterization using one-dimensional NMR spectroscopy: **Figure S2** S7
Section 5. Molecular characterization using ultrahigh resolution mass spectrometry (FT-ICR-MS): **Figures S3, S4** S9
Section 6. Distribution of Fe and Cu in wood and Fe oxidation state in relation to the Fenton chemistry: **Figures S5, S6** S12
Section 7. Effects of pressure-treatment on ConAC formation chemistry: **Table S2** S15
Section 8. Laboratory simulations of biomass oxidation: **Tables S3, S4; Figure S7** S17
References S20

---

[1] Present Address: State Key Laboratory of Marine Environmental Science, College of Ocean and Earth Sciences, Xiamen University, Xiamen City, Fujian Province, China, 361102

Section 1. Additional wood specimens (Pine 2 and Maple)

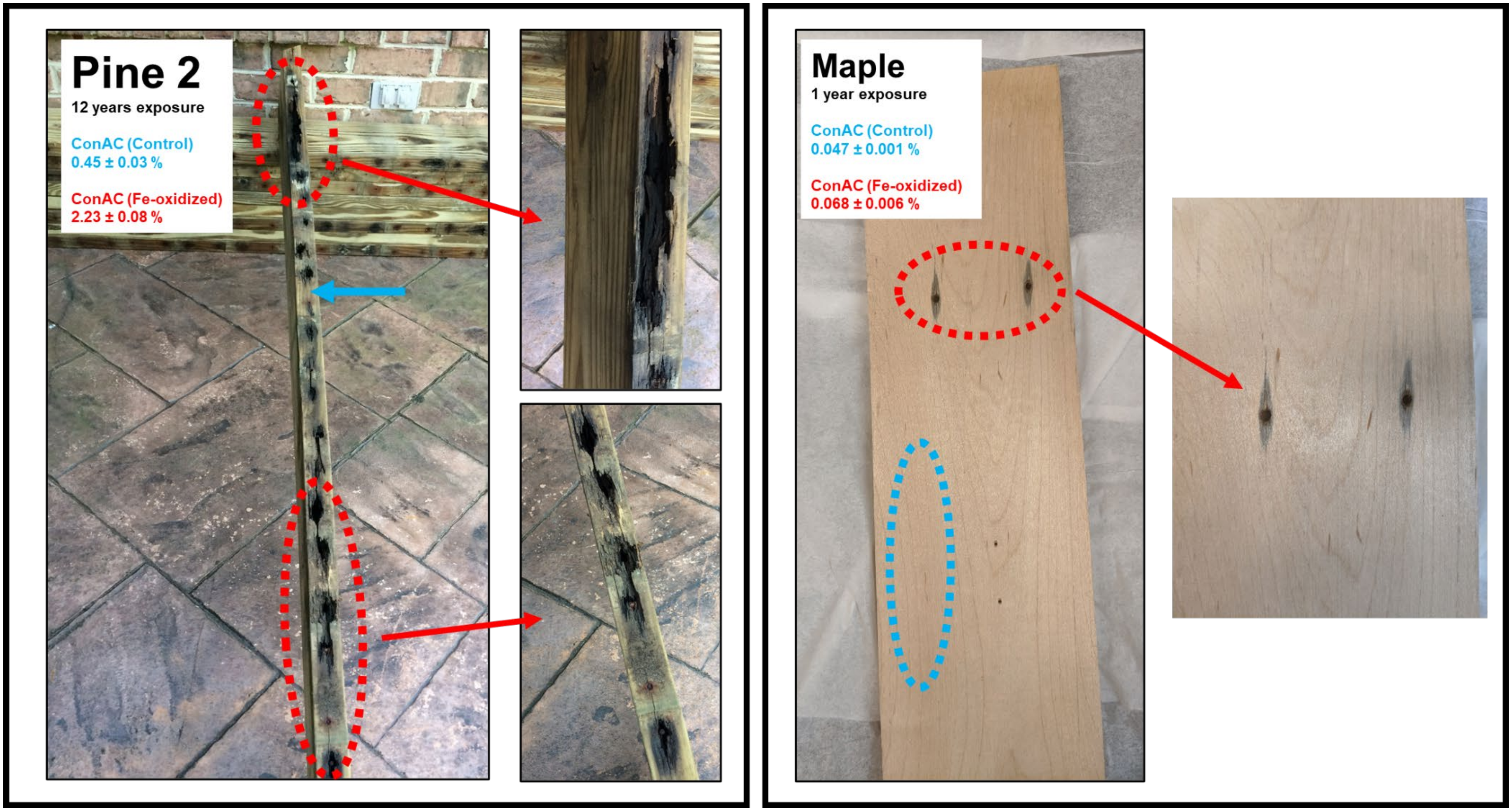

Figure S1. Charcoalification of pine (left) and maple (right) wood boards exposed to Fe nails. The **blue** arrow/circle indicate zones with no visible charcoalification, which were sampled to serve as representative controls. The **red** circles indicate zones with clear charcoalification. Condensed aromatic carbon (ConAC) quantities relative to organic carbon as well as lengths of exposure to nails are shown in the legends. The greenish tint of Pine 2 (left) is due to pressure treatment for retarding microbial degradation. Adapted with permission from Goranov.[1] Copyright ©, 2020, by Aleksandar Ivaylov Goranov, All Rights Reserved.

Section 2. Materials and methods for supplementary analyses

2.1. Quantification of organic carbon and hydrogen

Elemental analysis for carbon (C%) and hydrogen (H%) was performed using a Thermo Finnigan FlashEA 1112 elemental analyzer fitted with a CHN column. Samples were analyzed in triplicate and calibrated to a five-point external calibration curve of nicotinamide (CE Elantech, Inc.). Empty tin capsules were analyzed as blanks and to evaluate for any sample carryover. An aspartic acid standard (CE Elantech, Inc.) was also analyzed as a control sample to confirm the accuracy of the measurements. C% measurements were with relative standard deviations below 5%.

2.2. Quantification of metals

Metal (Cu% and Fe%) analysis was performed after ashing 20-30 mg solid samples at 600 ^{O}C in a temperature-controlled oven for 24 hours. The ash residue was then digested with 5 mL aqua regia (HNO_3:HCl = 1:3 molar ratio) for 12 hours, and the acidic mixture was evaporated in a sand bath at 70 ^{O}C. Then, the residue was resuspended in 5 mL 65% HNO_3 and the suspension was let to acid-digest for 6 hours, after which the acid was evaporated in a sand bath at 70 ^{O}C. Residue was resuspended in 5 mL 65% HNO_3 once more and HNO_3 was evaporated after the 6-hour digestion. Then, 5 mL 2% HNO_3 and ~1 mg of $La(NO_3)_3$ were added, and the nitrates formed during the acid digestions were dissolved with the assistance of a 10-minute sonication. The solutions were filtered using 0.22 uM 0.2 µm Teflon (PTFE) filters. Dissolved Fe and Cu were quantified using a Shimadzu AA-7000 atomic absorption spectrometer with a flame atomizer. Copper nitrate (1000 ppm, Acros Organics) and iron nitrate (10000 ppm, ASSURANCE) certified standards were used for external calibration. Instrument and procedural blanks were used to evaluate for contamination and sample carryover.

As it was known that the Pine 2 sample had been pressure-treated, its control and Fe-exposed samples were additionally assessed using inductively coupled plasma – mass spectrometry (ICP-MS). Samples were prepared as described above without the addition of $La(NO_3)_3$. Samples were analyzed on a Thermo Scientific Element XR double-focusing magnetic sector field ICP-MS instrument at the College of Sciences Major Instrumentation Cluster (COSMIC) facility at Old Dominion University (Norfolk, VA). The instrument was equipped with a perfluoroalkoxy alkane (PFA) microflow nebulizer and a quartz cyclonic spray chamber. Samples were taken up via an Elemental Scientific SC µ-DX autosampler with PFA probe, PFA sample tubing, and PFA sample vials. All ICP-MS front-end and sample input components were nitric-acid cleaned and sample probe was rinsed with 2% HNO_3 (matrix-matched) between samples. The Pine 2 Control and Fe-oxidized samples, along with a procedural blank, were screened for a variety of elements that are common in pressure-treatment agents (Al, As, B, Cd, Co, Cr, Cu, Fe, Mn, Ni, Pb, Sb, Si, Ti, V, Zn).[2] Concentrations were calculated by the Element XR software based on standard calibration curves. Computed calculations were manually double-checked afterwards. In addition to the procedural blank, a variety of other blanks were analyzed throughout the analytical sequence to verify for no background contamination. Metal concentrations were blank-corrected using the procedural blank and converted to weight percentages relative to sample weight (wt.%).

2.3. Solid-state nuclear magnetic resonance (NMR) analysis

Solid-state NMR analysis was performed on dried powdered samples, which were packed in a 4 mm Zirconia (ZrO_2) rotor with a polychlorotrifluoroethylene (Kel-F) cap. Analysis was done on a 400 MHz (9.4 Tesla) Bruker BioSpin AVANCE II spectrometer fitted with a 4 mm magic angle spinning (MAS) probe at the COSMIC facility. One-dimensional quantitative ^{13}C spectra were acquired using the quantitative multi-pulse cross-polarization MAS (MultiCPMAS) pulse program.[3] Samples were spun at the magic angle at 14 kHz and analyzed using a relaxation delay of 1 s, 5000 scans, 5 cross-polarization segments, and a total contact time of 3.30 ms. The obtained spectra were phased, calibrated to an external adamantane standard[4], and multiplied by an exponential window function (EM) of 50 Hz. Spectra were then baseline-corrected and integrated in the following ranges: Methyl: 0-20 ppm, Methylene: 20-45 ppm, O-Alkyl: 45-90 ppm, di-O-Alkyl: 90-110 ppm, Aryl: 110-146 ppm, Aryl-O: 146-165 ppm, Carboxyl/Ester (COO): 165-184, Carbonyl (CO): 184-220 ppm. All data were processed using the Bruker TopSpin 4.0.7. software.

2.4. X-ray fluorescence imaging and Fe X-ray absorption near edge structure (XANES)

Micro X-ray fluorescence (μ-XRF) images and micro X-ray absorption near edge structure (μ-XANES) spectra at the Fe K-edge were collected on the X-ray Fluorescence Microprobe (4-BM) beamline at the National Synchrotron Light Source II (NSLS-II, USA). The μ-XRF images were collected with an incidence X-ray energy of 13,500 keV at a step size of 10/40 μm. Sample fluorescence was detected using a 7-element Vortex detector and was integrated in the pre-determined regions of interest. Fe K-edge XANES spectra were measured with a step size of 0.3 eV at the absorption edge and were aligned using a reference spectrum of Fe foil (first inflection point of the edge set at 7,112.0 eV). A focused beam (~ 10 × 10 μm^2) was used throughout the experiment.

Image processing and data analysis were performed in Larch and R. Energy alignment, and XANES spectral normalization were conducted using Athena. Energies of pre-edge features were determined using 2nd derivative spectra in the GRAMS software. Pre-edge centroids were calculated as intensity-weighted means of pre-edge peak energies and compared with literature values.[5] All data have been corrected by applying a 0.9 eV energy shift because of a difference in calibrating Fe foil energy.

2.5. Extraction of organic matter

Base-extraction was selected as the method for obtaining a representative liquid extract of the solid control and Fe-oxidized wood samples. The extraction was performed using 0.1 M sodium hydroxide (Fisher, ACS Certified grade) at a ratio of 0.5 g sample/100 mL extractant. Suspensions were vigorously stirred on a shaker table for 24 hours. Then, the supernatant was removed and substituted with new 100 mL of extractant. The extraction was done three times over 3 x 24 hours to result in a total of 300 mL base-extract of each sample. Base-extracts were then filtered through pre-combusted 0.7 μm glass-fiber filters (GF/F, Whatman, 47 mm diameter) and cation-exchanged using a Dowex 50Wx8 resin (Acros Organics). A procedural blank of sodium hydroxide was processed the same way. All extractions were performed under inert (N_2) atmosphere. The procedure followed the International Humic Substances Society (IHSS) guidelines and is evaluated and described in greater detail elsewhere.[6]

2.6. Ultrahigh resolution mass spectrometry (FT-ICR-MS)

Cation-exchanged base-extracts were diluted to 50 mg/L carbon-equivalents (50 $mgC \cdot L^{-1}$) and then further diluted with methanol (CH_3OH, Fisher Scientific, Optima LC-MS grade) to give 1:1 $CH_3OH:H_2O$ mixtures. Samples were analyzed on a Bruker Daltonics 12-Tesla Apex Qe FT-ICR-MS housed in the COSMIC facility. The instrument was calibrated daily with a polyethylene glycol standard and instrument blanks were analyzed in-between samples to assure for no sample carryover. Samples were infused into the Apollo II electrospray ionization (ESI) source at flow rate of 120 µL/h and molecules were ionized in negative mode. Ionization voltages were optimized on a per-sample basis to assure for uniform spray currents across the dataset. The ionized molecules were collected in a hexapole, filtered by a quadrupole for a mass range of 200-1200 m/z, pre-concentrated in a second hexapole, and transferred into the ICR cell where 300 transients were collected. They were co-added, and the resultant free induction decay was zero-filled and sine-bell apodized. After a fast Fourier transformation, spectra were calibrated to naturally abundant fatty acids, dicarboxylic acids, and compounds belonging to the CH_2-homologous series.[7] Peaks with signal-to-noise above 3 were exported to MATLAB where salt, blank, and ^{13}C isotopologue peaks were removed from each spectrum. Molecular formulas were assigned to each mass list using the Molecular Formula Calculator from the National High Magnetic Field Laboratory (Tallahassee, FL). Formulas were restricted to elemental composition of $^{12}C_{5-\infty}$, $^{1}H_{0-100}$, $^{16}O_{0-50}$, $^{14}N_{0-10}$, $^{32}S_{0-4}$, and $^{31}P_{0-2}$, and the obtained formulas were refined following previously published criteria.[8-10] No ambiguous assignments were left in the final formula lists (i.e., for each mass spectral peak there was only one molecular formula). Only formulas containing carbon, hydrogen, and oxygen elements are used hereafter (i.e., CHO formulas).

Molecular formulas are further classified based on their modified aromaticity index (AI_{MOD}), a measurement of the double-bond density in a molecule.[11, 12] Compounds with $AI_{MOD} = 0$ are classified as “aliphatic”. Molecules with $0 < AI_{MOD} < 0.5$ have either an aromatic moiety that is highly functionalized with aliphatic groups or have olefinic/alicyclic bonds. Molecules with $0.5 \le AI_{MOD} < 0.67$ are classified as aromatic. Formulas with $AI_{MOD} \ge 0.67$ and number of C-atoms ≥ 15 are classified as condensed aromatic.[11-13] The calculation for this index is shown below.

$$AI_{MOD} = \frac{1 + C - \frac{1}{2}O - S - \frac{1}{2}(N + P + H + Cl)}{C - \frac{1}{2}O - N - S - P}$$

Data analysis was performed using codes of the MATLAB-based Toolbox for Environmental Research (TEnvR).[14]

Section 3. Bulk elemental characteristics

Bulk elemental characterization revealed an enrichment in carbon and loss of hydrogen (Table S1), which resulted in the decrease of H/C ratios for the three specimens. This is indicative of forming aromatic carbon following exposure to Fenton chemistry (Figure 2). Solid-state ^{13}C NMR also confirmed the formation of aromatic carbon by showing an increase in aryl and phenolic (Aryl-O) functional groups (49 – 98%, Figure S2). The increase in aromatic content is partially responsible for the darkening of the wood adjacent to each nail. In addition to phenolic groups, one- to two-fold increases in ketone (CO) and carboxyl (COO) groups are found (Figure S2), which are indicative of oxidation and are expected for these Fe-wood systems. Mass spectrometric analysis of alkaline extracts of the two pine samples showed a shift of the molecular composition towards a higher O/C ratio (Figure S4), which is a clear indication that the charcoalified materials had experienced oxidation relative to their control biomass. The observed changes in the organic matter composition show great similarity to what one would observe from plant litter being degraded in the environment[15, 16]. We further confirmed the involvement of Fenton chemistry by examining the Fe oxidation state using synchrotron X-ray absorption near-edge fine structure (XANES). The presence of abundant oxidized Fe^{III} in the wood samples adjacent to the Fe nails is consistent with the action of Fenton oxidation (Figures S5-S6).

Table S1. Elemental analysis and benzenepolycarboxylic acids (BPCA) quantification of ConAC in the two pine wood samples. LOD = limit of detection

Sample	C (wt.%)	H (wt.%)	H/C (mol/mol)	Fe (wt.%)	Cu (wt. %)	ConAC/OC (wt. %)	Annual ConAC production (%/year)*
Pine 1 Control	46.1 ± 0.4	6.0 ± 0.1	1.55 ± 0.03	Below LOD	0.026 ± 0.002	0.46 ± 0.01	Not Applicable
Pine 1 Fe-exposed	47.0 ± 2.0	5.2 ± 0.2	1.33 ± 0.03	4.7 ± 0.2	0.050 ± 0.003	2.44 ± 0.05	0.203 %/year
Pine 2 Control	46.2 ± 0.6	5.8 ± 0.2	1.49 ± 0.06	Below LOD	0.71 ± 0.07	0.45 ± 0.03	Not Applicable
Pine 2 Fe-exposed	50.1 ± 0.6	5.2 ± 0.1	1.23 ± 0.03	4.4 ± 0.7	0.34 ± 0.03	2.23 ± 0.08	0.186 %/year
Maple Control	48.0 ± 0.4	6.5 ± 1.1	1.62 ± 0.28	Not Determined	Not Determined	0.047 ± 0.001	Not Applicable
Maple Fe-exposed	46.2 ± 0.3	5.7 ± 0.1	1.47 ± 0.2	Not Determined	Not Determined	0.068 ± 0.006	0.068 %/year

*Annual ConAC production is calculated over 12 years of Fe-exposure for the two pine boards and over 1 year of exposure of the maple board. It is expressed as percentage relative to the organic carbon (OC). ConAC in the controls is not included in the calculation, because fresh wood is ConAC-free.[17] The average biomass-to-ConAC conversion (**0.195 %/year**) was computed by averaging the rates derived from the two pine boards (0.203, 0.186 %/year) .

Section 4. Structural characterization using one-dimensional NMR spectroscopy

Bulk structural characteristics of the two wood samples before and after exposure to Fenton chemistry were determined using one-dimensional nuclear magnetic resonance (NMR) spectroscopy (Figure S2). Solid-state ^{13}C NMR analysis is a classical approach for evaluating solid environmental matrices[18, 19], and utilizing the multi-pulse cross-polarization magic angle spinning ^{13}C NMR technique (MultiCPMAS ^{13}C NMR) allows for quantitative reporting of the total content of the various ^{13}C functionalities in the studied samples. The obtained MultiCPMAS ^{13}C NMR spectra for the control samples were highly characteristic for woody samples.[20] Lignin and carbohydrate signatures are easily identified and seem to predominate in the spectra (left panels of Figure S2), as these biopolymers are of the highest abundance in woody biomass, carbohydrates being the most abundant and lignin being the second most abundant biopolymer, respectively.[21] Lignin's presence is identified by the peaks associated with its methoxy ($\mathbf{C}H_3O$-, 56 ppm) and phenolic ($\mathbf{C}_{\mathbf{Aryl}}$-O, 147 ppm) functionalities. Cellulose's glycosidic units present seven peaks in the area between 60 and 110 ppm (C_1-C_6,). There are several peaks in the region of 0-45 ppm, where methyl ($\mathbf{C}H_3$-) and methylene (-$\mathbf{C}H_2$-) functionalities usually resonate. These are generally associated with acetylated glucose units in hemicelluloses and resinous substances. Pine 2 is a pressure-treated wood, thus its aliphatic resonances could also originate from alkyl groups in the quaternary ammonium ligands of commonly used pressure-treatment reagents.[2] The peak at 172 ppm is associated with carboxyl groups (-**C**OOH) or derivatives (-**C**OOR) such as amides or esters, and this peak likely corresponds to acetyl esters of hemicellulose, cellulose lactones, and carboxyl groups of gluconic and glucuronic acids of oxidized cellulose.

Nearly all peaks found in the Fe-oxidized samples are present in the control samples (left panels of Figure S2), which is expected as both samples are from the same parent source. The distribution (peak intensity) of carbon moieties has changed after the decade-long Fenton oxidation – carbonyl, carboxyl, phenolic, and aromatic groups increase (right panels of Figure S2) showing that the exposure to Fe in the nail over decade-long exposure period has enriched the two Fe-oxidized samples in aromatic structures. This appears to be at the expense of cellulosic materials, as is evident by the diminution of di-O-alkyl and O-alkyl groups. Carbohydrates are labile towards oxidative processes and their degradation pathways via the Fenton reaction have been previously studied[22, 23], which explains the presented data here. It must be noted that the abundance of Fe in the Fe-oxidized samples (and copper in the Pine 2 samples) may have quenched some of the aromatic signals[24, 25], thus the abundance of aromatic carbons in the Fe-oxidized samples is likely much higher.

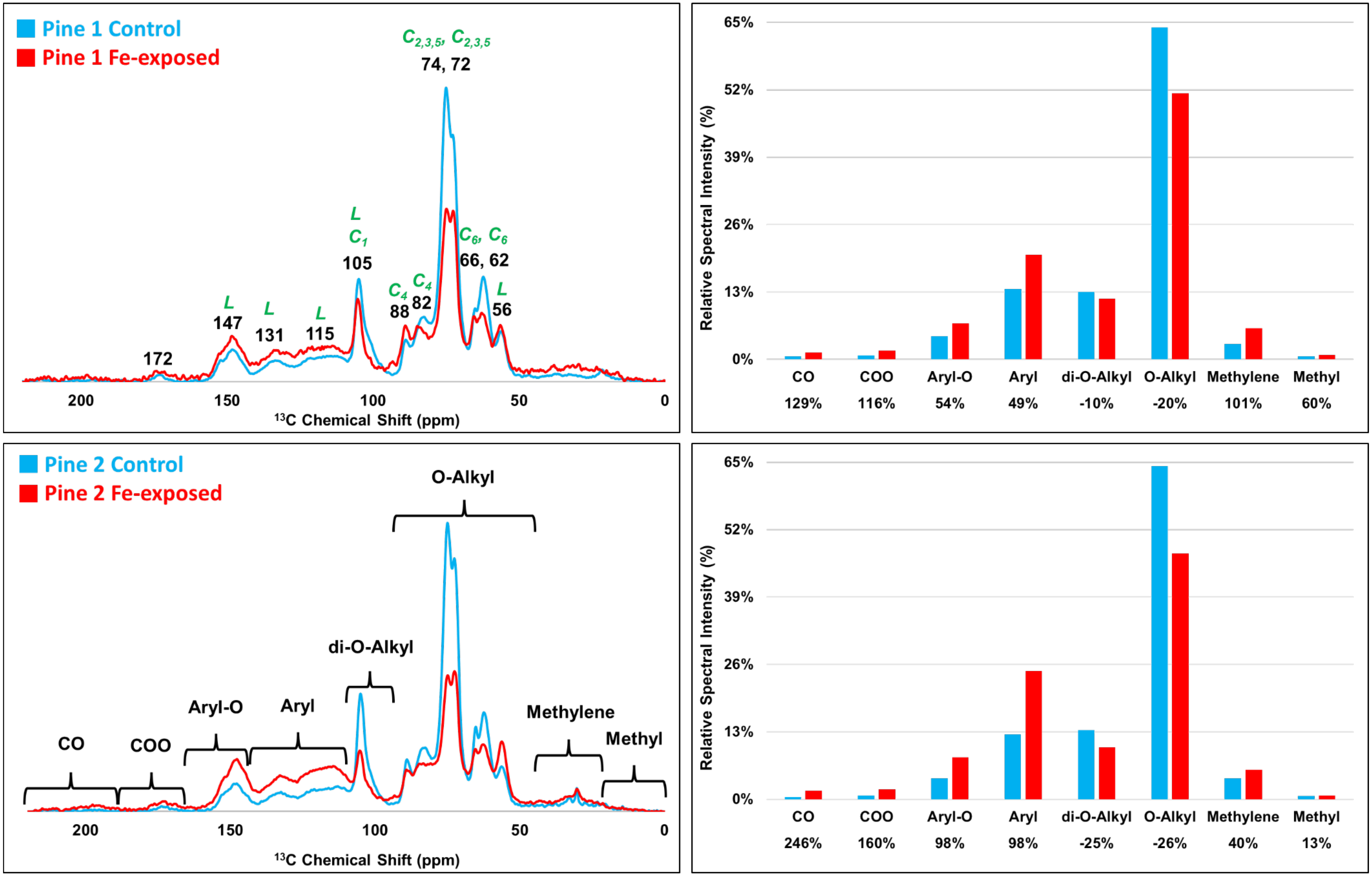

Figure S2. One-dimensional solid-state ^{13}C NMR analysis of whole wood samples (Control colored in **blue** and Fe-oxidized in **red**). Left panels show the NMR spectra, with labeled peaks (top) and chemical functionalities (bottom panel). Carbohydrate and Lignin resonances, along with the corresponding carbon numbers, are labeled in **green** (***C*** and ***L***, respectively). Right panels show the integrated chemical shift regions, with percent change in functionality abundance shown under each label in the x-axis. Adapted with permission from Goranov.[1] Copyright ©, 2020, by Aleksandar Ivaylov Goranov, All Rights Reserved.

Section 5. Molecular characterization using ultrahigh resolution mass spectrometry (FT-ICR-MS)

To investigate the 12-year exposure to Fenton chemistry on the molecular level, ultrahigh resolution mass spectrometry was employed. Given that the utilized FT-ICR-MS instrument did not have an ionization source suitable for analyzing solid samples, the four samples of this study were analyzed after an alkali extraction, a classical approach from the soil sciences. Though a large fraction of the extracted sample remains as a solid residue (known as humin), the base-extract is usually representative of the structural composition of the evaluated sample.[6] To confirm this for our samples, we employed solid-state NMR analysis on the freeze-dried base-extracts and compared them with the original solid samples (Figure S3). The base-extracts contained all functional groups native to the whole samples concluding that our base-extracts were representative. A large portion of the cellulose was not extracted (O-alkyl and di-O-alkyl peaks at 62-105 ppm), however, carbohydrates do not ionize efficiently in the ESI source, and are therefore barely covered by the analytical window of the ESI-FT-ICR-MS. Thus, their lower amount in the base-extracts was not of serious concern.

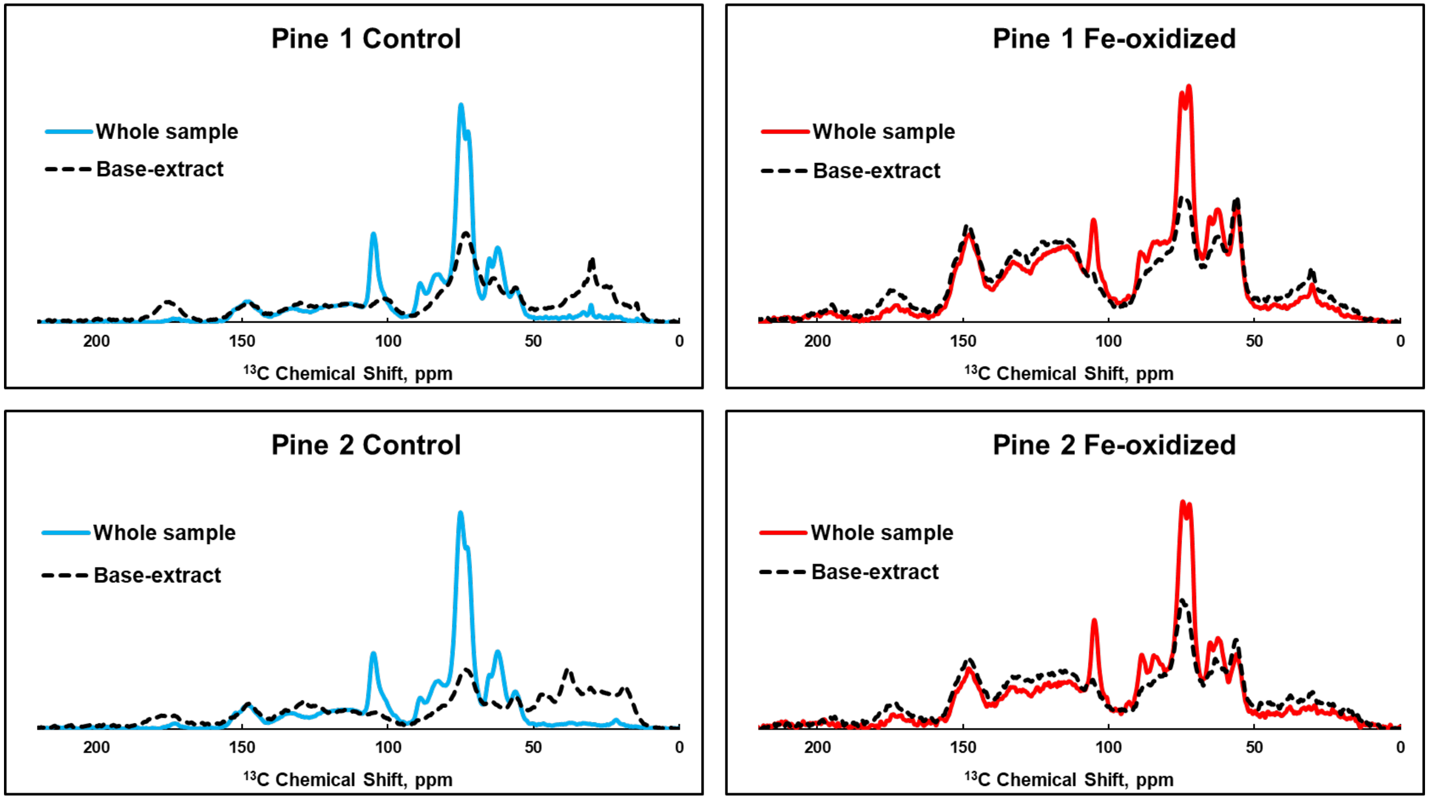

Figure S3. Solid-state ^{13}C NMR spectra of whole samples (in **blue** or **red**) relative to freeze-dried base-extracts (in **black**).

The employed FT-ICR-MS technique has become extremely popular in organic geochemistry as it is able to resolve the thousands of molecules in complex environmental samples and evaluate their composition.[26-29] While this instrument only measures mass-to-charge values for all ionized molecules in a sample, its ultrahigh precision allows for a unique molecular formula to be assigned to each mass peak. While not quantitative, it is another common tool for detecting the presence of ConAC in various samples.[30, 31] Similar to the assumption employed for the BPCA method (i.e., quantified ConAC is labeled as pyrogenic BC), formulas with modified aromaticity index[11, 12] $AI_{MOD} \geq 0.67$ corresponding to ConAC are often labeled as BC[10, 32, 33], and if they contain a N or S atom, they are even referred to as black nitrogen[34] and black sulfur[35], respectively. The assigned molecular formulas to Pine 1 and 2 controls and Fe-oxidized samples are classified using a presence/absence approach[36] in three categories: Fenton-labile (formulas present in the control samples); Fenton-resistant (formulas present in both samples); and Fenton-produced (formulas present in the Fe-oxidized sample). This approach allows one to observe what molecular features were produced upon the Fenton oxidation of wood (Figure S4).

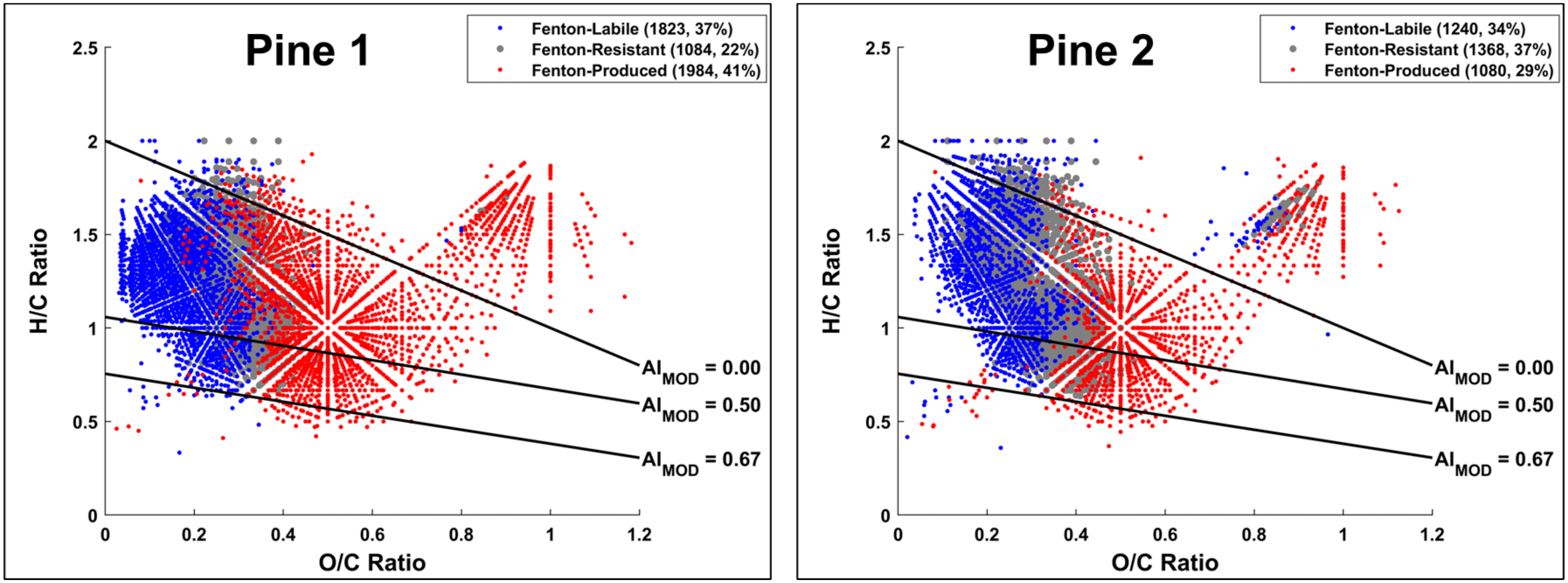

Figure S4. Van Krevelen diagrams (H/C vs. O/C) of FT-ICR-MS formulas identified only in the control (**Fenton-Labile**), only in the Fe-oxidized (**Fenton-Produced**), or in both samples (**Fenton-Resistant**) using a presence/absence approach.[36] The number of formulas of each class (and corresponding percentages) are given in parentheses in the legends. The van Krevelen space is separated based on modified aromaticity index (AI_{MOD}) thresholds.[11, 12] Reprinted with permission from Goranov.[1] Copyright ©, 2020, by Aleksandar Ivaylov Goranov, All Rights Reserved.

After exposure to Fenton chemistry there are clear shifts in the molecular composition of both woods. For both samples, molecular formulas with higher O/C ratio and lower H/C ratio evolve, which is also accompanied by loss of numerous aliphatic/olefinic compounds (blue markers). These compounds are likely carboxyl-containing aliphatic molecules recently found and proposed to be important in the formation process of aromatic compounds, including ConAC, in soils.[37, 38] The pools of Fenton-produced molecules contain lignin-like and carbohydrate-like formulas, as well as some ConAC formulas falling under the $AI_{MOD} \geq 0.67$ line (Figure S4). If these data are interpreted using the traditional approaches in the wildfire literature[10, 32-35], it can be concluded that ConAC is produced after Fenton exposure. This data complements the trends presented using BPCA analysis and are in agreement with the previously published non-pyrogenic pathways for formation of ConAC from ligninaceous molecules.[39, 40] The observed changes are also consistent with humification reactions in soils[15, 16], which are known to produce aromatic and condensed aromatic structures.[41, 42] This observation also gives more merit to the previously made hypothesis that soil humics are derived from lignin after processing via radical electrocyclization reactions.[38] However, a more in-depth analysis of these samples using humic extractions must be done to directly test this proposition. A recent study, which determined that microbe-induced humification produces condensed moieties[43], is also in agreement with what is presented here in which Fenton exposure leads to humification and formation of ConAC. The molecular results here also parallel with the quantitative structural data from solid-state ^{13}C NMR data (Figure S2), further validating our proposition of oxidative chemistry driving the chemical composition and formation of non-pyrogenic ConAC.

A change in the molecular composition of carbohydrates is also evident. Wood contains mainly cellulosic carbohydrates[44], which are known to be labile towards the Fenton reaction.[22, 23] It is likely that the Fenton-produced formulas (blue markers) are of gluconic and glucuronic acids, products of the oxidative degradation of cellulose. Such transformation is consistent with oxidative degradation of cellulose in the environment.[45-47]

Section 6. Distribution of Fe and Cu in wood and Fe oxidation state in relation to the Fenton chemistry

The distribution of Fe and Cu in two wood cross-sections (73 × 1 mm^2, 20 × 2 mm^2), starting from the Fe-exposed area to the adjacent unaffected “control” area, were evaluated using micron-sized synchrotron X-ray beams (Figure S5a). The abundance of Cu follows the dark and light growth bands of wood with alternating Cu-rich and Cu-poor regions (Figure S5b) suggesting that these bands originated from the pressure-treatment. In contrast, Fe is present in patches of high-abundance hotspots in addition to the normal patterning that follows tree growth bands (Figure S5b) suggesting that the non-hotspot Fe distribution is intrinsic to the initial wood material. The Cu abundance in the Fe-exposed area is less when compared to the unaffected area (Figure S5c) indicating leaching of material over the time of exposure.[48, 49] The abundance of Fe is greater in the nail-exposed area, with a two-fold increase in Fe fluorescence intensity (0.014 to 0.031, Figure S5c). This increase in Fe abundance around the Fe-nail is likely from oxidative leaching by Fe from the nail, and diffusion and accumulation in the wood.[49] Our interpretation for leaching and diffusion is also supported by the observation of distinct random patches throughout the “control” areas of the wood suggesting that Fe(II) and Fe(III) species diffused throughout time and possibly precipitated as distinct Fe minerals (oxides, hydroxides, and/or oxyhydroxides) or Fe-ConAC complexes.[50]

To evaluate changes in the Fe oxidation state and examine the role of Fe in altering the organic matter, Fe K-edge XANES spectra were evaluated at randomly selected locations in both Fe-oxidized and control areas of the wood. The pre-edge peak maximum, indicative of Fe-oxidation state[5], appears between 7,113 – 7,115 eV (Figure S5d). These peak maxima correspond to two types of Fe species that are found across the Fe exposure continuum: one being a mixture of Fe(II) and Fe(III), and the other being close to pure Fe(III). While the Fe abundance varies significantly among the Fe-oxidized and control regions, pre-edge energies of Fe in the two regions are not statistically different ($p = 0.98$). Additionally, a great variation in Fe oxidation state is observed within a few millimeters around the Fe-oxidized region when compared to the background “control” area of the wood. This large variation in Fe oxidation states has likely resulted from the Fenton chemistry, in which Fe is actively oxidized and reduced, leaving both Fe(II) and Fe(III) species in the wood. Such constant cycling of redox Fe speciation has been shown to be a key driver in the cycling of organic matter in soils.[51]

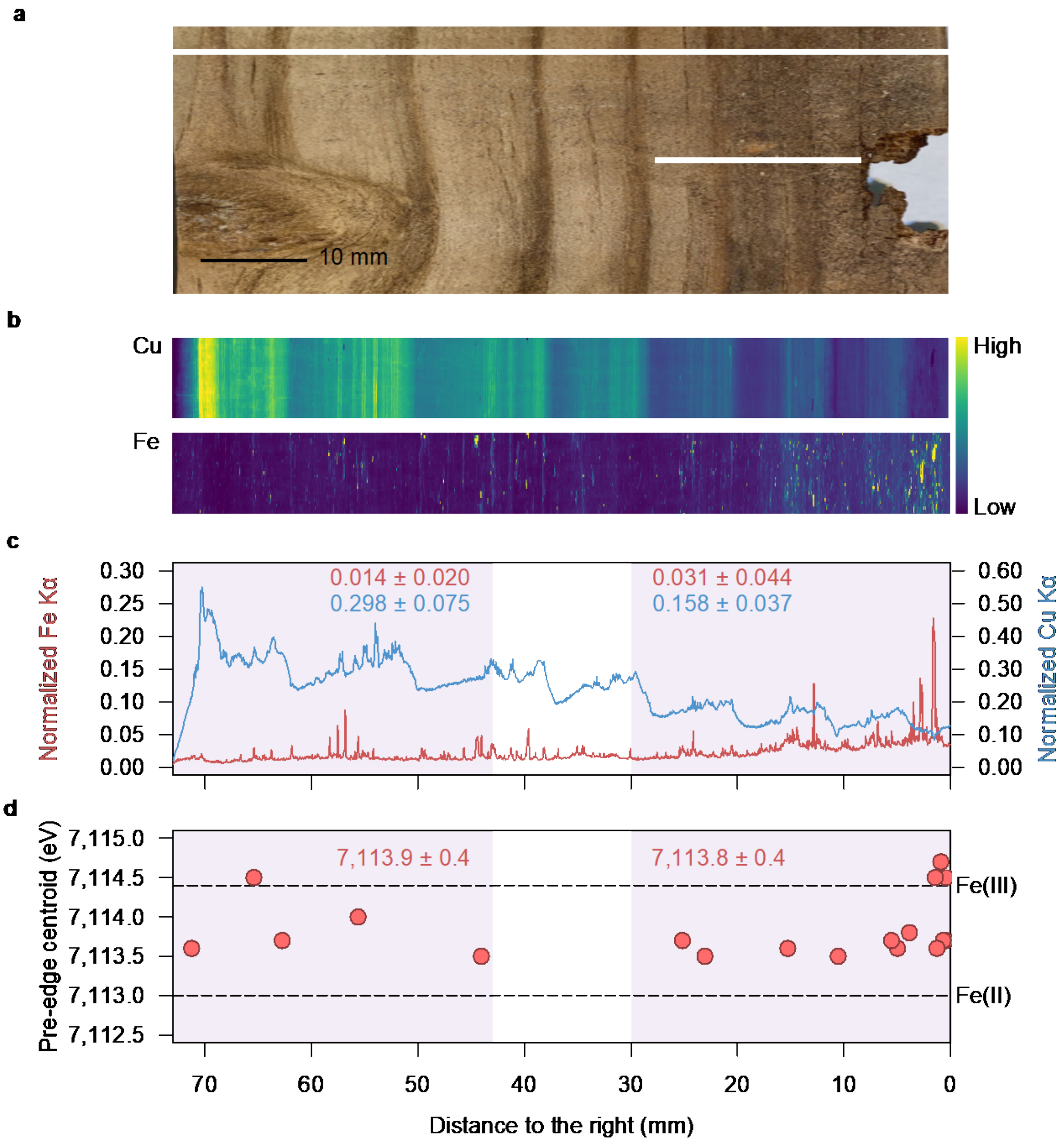

Figure S5. Cu and Fe distribution and Fe speciation in Pine 2. a) A photograph of the imaged sample is shown on the top with the darkened Fe-oxidized area in the right. X-ray imaged cross-sections are labeled with white bars. Images and data for the short section can be seen in Figure S6; b) XRF elemental maps of Cu and Fe; c) Plot of integrated Fe (**red**) and Cu (**blue**) Kα intensities across the long section. The mean and standard deviation of for Kα intensity for each of the shaded 30-mm zones are shown as well; d) Plot of centroid position of pre-edge peaks of Fe K-edge XANES spectra across the imaged sample, with the means and standard deviations for each 30-mm zone. Reference lines of Fe(II) and Fe(III) pre-edge centroids are from previously published measurements.[5] Note that the images in a) and b) are compressed and elongated in the vertical direction, respectively, for clarity.

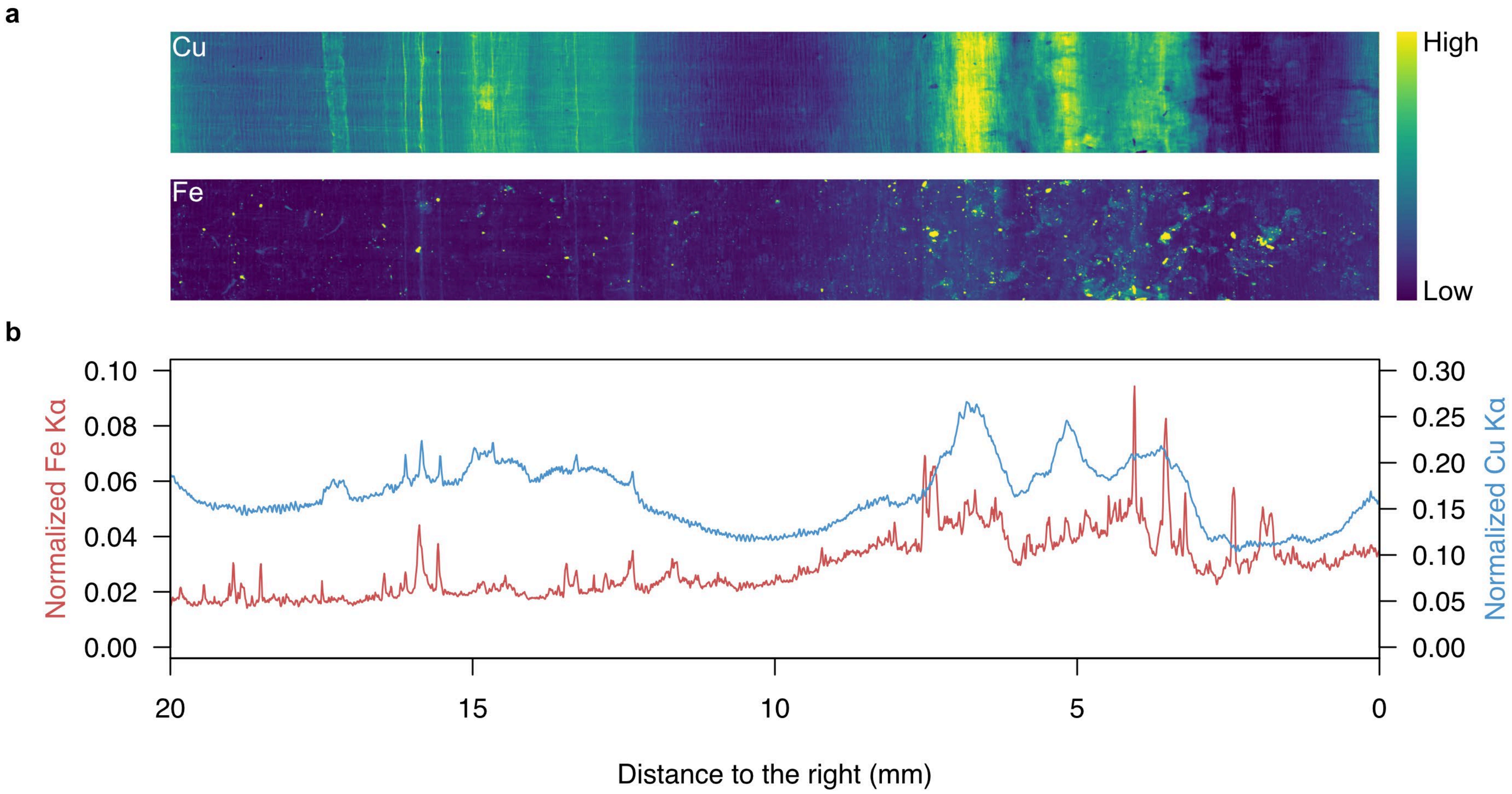

Figure S6. Cu and Fe elemental distribution in the short section from Pine 2 (see Figure S5): a) Cu and Fe Kα images with the color bar showing the relative intensity; b) Integrated Fe and Cu Kα intensities on this section.

Section 7. Effects of pressure-treatment on ConAC formation chemistry

The Pine 2 boards had been pressure-treated, which meant that they contained a preservative within their chemical composition. The Pine 2 Control and Fe-oxidized samples were screened using ICP-MS for a variety of elements that are common in pressure-treatment agents (Al, As, B, Cd, Co, Cr, Cu, Fe, Mn, Ni, Pb, Sb, Si, Ti, V, Zn).[2] Only Cu was found in significant amounts (Table S2). This allowed us to conclude that Pine 2 had been treated with a Cu-containing preservative. Such compounds usually contain alkylated quaternary ammonium ligands[2], which would be responsible for the unusual methylene (<u>**C**</u>H_2) residues in this sample that are not found in Pine 1 (Figure S2).

One may argue that the preservative used is responsible for the observed formation of ConAC in Pine 2. Perhaps a Cu-driven Fenton oxidation[52] was occurring throughout the boards and caused ConAC production. However, the close association of the charcoalification to emplaced Fe nails indicates that Fenton reactions driven by Fe were dominant (Figure S1). However, a Cu-driven Fenton oxidation could explain the existence of small amounts of ConAC in the Control sample of Pine 2. By comparing trends for Pine 1 and Pine 2 from all analytical techniques it can be observed that the samples show very similar trends in their Fenton-driven structural compositions. This indicates that the pressure-treatment preservative in Pine 2 is highly refractory to Fenton-chemistry and does not participate in the transformation of organic matter nor in the production of ConAC. This makes sense given that the common Cu-containing preservatives contain long aliphatic chains, which are compounds lacking olefinic bonds and thus, being unable to participate in Diels-Alder-like cyclization reactions.[39]

The availability of a pressure-treated sample in this dataset allowed us to have a sample where microbial degradation had been quenched. Microbial degradation of lignin has been shown to produce ConAC[43], which could have occurred in Pine 1, but the pressure-treatment of Pine 2 quenched such processes and the ConAC formation in Pine 2 was likely abiotic in its entirety. The high similarity of data among Pine 1 and Pine 2 suggests that Fenton-driven formation of ConAC is more prominent than microbiological ConAC formation for Pine 1, additionally giving merit to our perspective that oxidation chemistry is an integral part of the non-pyrogenic ConAC formation in soils and other systems.

Table S2. Screening of Pine 2 using inductively couple plasma – mass spectrometry for the presence of elements typically contained in pressure-treatment chemicals. All results are reported as parts per million (ppm) of metal relative to sample weight.

	**Pine 2 Control**	**Pine 2 Fe-exposed**
**Al (ppm)**	88 ± 9	112 ± 11
**As (ppm)**	0.33 ± 0.03	0.32 ± 0.03
**B (ppm)**	0.042 ± 0.004	0.022 ± 0.002
**Cd (ppm)**	0.007 ± 0.001	0.043 ± 0.004
**Co (ppm)**	1.1 ± 0.1	0.9 ± 0.1
**Cr (ppm)**	8.6 ± 0.9	1.3 ± 0.1
**Cu (ppm)**	**7101 ± 726**	**3447 ± 352**
**Fe (ppm)**	**159 ± 16**	**6201 ± 634**
**Mn (ppm)**	11 ± 1	76 ± 8
**Pb (ppm)**	0.9 ± 0.1	1.7 ± 0.2
**Sb (ppm)**	0.013 ± 0.001	0.028 ± 0.003
**Ti (ppm)**	3.0 ± 0.3	3.1 ± 0.3
**V (ppm)**	0.23 ± 0.02	0.21 ± 0.02
**Zn (ppm)**	11 ± 1	186 ± 19

Section 8. Laboratory simulations of biomass oxidation

Table S3. Quantities of carbon and condensed aromatic carbon (ConAC) at different oxidation time points for the experiments with different biomass (Figure 3)

	**C at start of oxidation, mg**	**C at end of oxidation*, mg**	**ConAC at start of oxidation, mg**	**ConAC at end of oxidation**, mg**	**ConAC produced, mg**	**Biomass-to-ConAC conversion**
**Maple Wood Day = 1**	15.2 ± 0.8	14.6 ± 0.7	0.010 ± 0.001	0.273 ± 0.014	0.262 ± 0.013	**1.73 ± 0.09 %**
**Maple Wood Day = 2**	17.5 ± 0.9	12.6 ± 0.6	0.012 ± 0.001	0.445 ± 0.022	0.433 ± 0.022	**2.46 ± 0.12 %**
**Corn Root Day = 1**	15.6 ± 0.8	7.4 ± 0.4	0.083 ± 0.004	0.164 ± 0.008	0.081 ± 0.004	**0.52 ± 0.03 %**
**Corn Root Day = 2**	14.8 ± 0.7	6.1 ± 0.3	0.079 ± 0.004	0.228 ± 0.011	0.149 ± 0.007	**1.00 ± 0.05 %**
**Lignin Concentrate Day = 1**	21.0 ± 1.1	16.8 ± 0.8	0.659 ± 0.033	0.885 ± 0.044	0.226 ± 0.011	**1.07 ± 0.05 %**
**Lignin Concentrate Day = 2**	21.4 ± 1.1	15.1 ± 0.8	0.671 ± 0.034	1.145 ± 0.057	0.475 ± 0.024	**2.22 ± 0.11 %**
**Pine Wood (Pine 1) Day = 1**	18.2 ± 0.9	19.1 ± 1.0	0.172 ± 0.009	0.235 ± 0.012	0.063 ± 0.003	**0.34 ± 0.02 %**
**Pine Wood (Pine 1) Day = 2**	16.7 ± 0.8	18.3 ± 0.9	0.158 ± 0.008	0.401 ± 0.020	0.243 ± 0.012	**1.45 ± 0.07 %**
**Dried Algae Day = 1**	11.9 ± 0.6	10.8 ± 0.5	0.055 ± 0.003	0.083 ± 0.004	0.028 ± 0.001	**0.23 ± 0.01 %**
**Dried Algae Day = 2**	10.2 ± 0.5	4.7 ± 0.2	0.047 ± 0.002	0.106 ± 0.005	0.058 ± 0.003	**0.57 ± 0.03 %**
**Bark Day = 1**	22.6 ± 1.1	22.8 ± 1.1	0.516 ± 0.026	1.341 ± 0.067	0.825 ± 0.041	**3.64 ± 0.18 %**
**Bark Day = 2**	21.7 ± 1.1	24.5 ± 1.2	0.495 ± 0.025	2.258 ± 0.113	1.763 ± 0.088	**8.11 ± 0.41 %**

*corrected for extraneous carbon inputs from the H_2O+HCl+$FeSO_4$+H_2O_2 reagents

**there were no extraneous ConAC inputs (procedural blanks were ConAC-free)

Table S4. Quantities of carbon and ConAC at different points for the harsh oxidation experiment of maple wood (Figure 4)

Oxidation time, days	C at start of oxidation, mg	C at end of oxidation*, mg	ConAC at start of oxidation, mg	ConAC at end of oxidation**, mg	ConAC produced, mg	Biomass-to-ConAC conversion
**0.236**	46.2 ± 2.3	23.0 ± 1.1	0.032 ± 0.002	0.095 ± 0.005	0.063 ± 0.003	**0.136 ± 0.007 %**
**0.740**	46.0 ± 2.3	32.5 ± 1.6	0.032 ± 0.002	0.131 ± 0.007	0.100 ± 0.005	**0.217 ± 0.011 %**
**1.799**	46.0 ± 2.3	29.9 ± 1.5	0.032 ± 0.002	0.114 ± 0.006	0.082 ± 0.004	**0.178 ± 0.009 %**
**3.986**	46.1 ± 2.3	9.3 ± 0.5	0.032 ± 0.002	0.069 ± 0.003	0.037 ± 0.002	**0.081 ± 0.004 %**
**4.729**	45.8 ± 2.3	4.2 ± 0.2	0.031 ± 0.002	0.074 ± 0.004	0.043 ± 0.002	**0.093 ± 0.005 %**
**5.111**	46.2 ± 2.3	2.0 ± 0.1	0.032 ± 0.002	0.065 ± 0.003	0.033 ± 0.002	**0.072 ± 0.004 %**
**7.792**	46.1 ± 2.3	3.2 ± 0.2	0.032 ± 0.002	0.059 ± 0.003	0.027 ± 0.001	**0.058 ± 0.004 %**
**10.146**	45.9 ± 2.3	4.4 ± 0.2	0.032 ± 0.002	0.059 ± 0.003	0.027 ± 0.001	**0.059 ± 0.003 %**

*corrected for extraneous carbon inputs from the H_2O+HCl+$FeSO_4$+H_2O_2 reagents

**there were no extraneous ConAC inputs (procedural blanks were ConAC-free)

The baseline value estimating biomass conversion to refractory ConAC (**0.063 ± 0.008 %**) was calculated by averaging the last three time points (0.072, 0.058, 0.059) and computing the associated standard deviation.

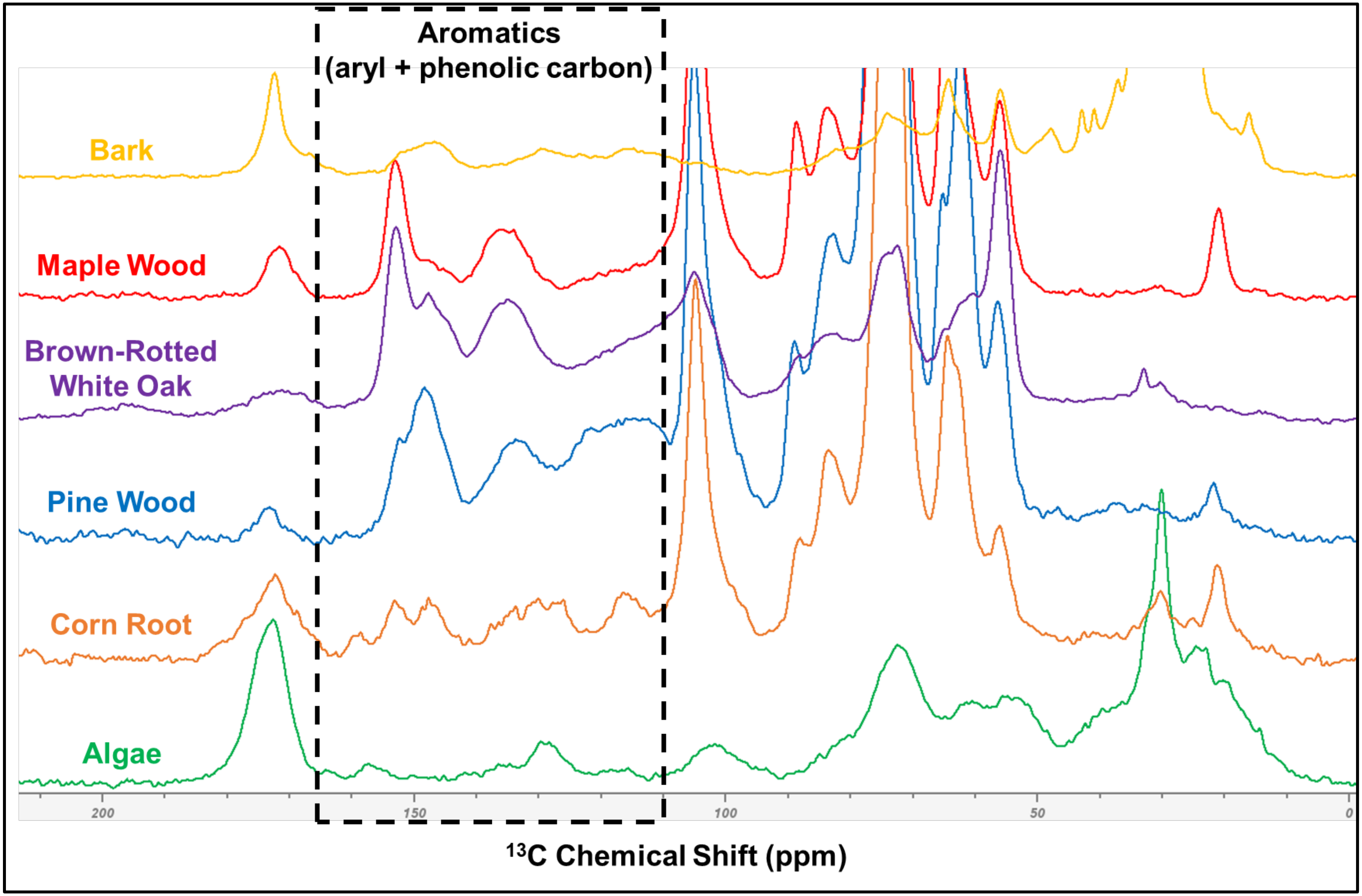

Figure S7. Solid-state ^{13}C NMR spectra of biomass materials used in laboratory oxidations. Boxed is the region of aromatics where aryl (110 – 146 ppm) and phenolic (146 – 165 ppm) functional groups resonate. These correspond to phenols in lignin and tannins, monoaromatic groups in suberin, as well as aromatic rings in proteins.

References

(1) Goranov, A. I. Advances in the understanding of sourcing and fate of pyrogenic organic matter in the environment. Doctoral Dissertation, Old Dominion University, Norfolk, VA, 2020. DOI: 10.25777/fpsv-4e28

(2) Freeman, M. H.; McIntyre, C. A comprehensive review of copper-based wood preservatives with a focus on new micronized or dispersed copper systems. *Forest Products Journal* **2008**, *58*, 6-27.

(3) Johnson, R. L.; Schmidt-Rohr, K. Quantitative solid-state ^{13}C NMR with signal enhancement by multiple cross polarization. *Journal of Magnetic Resonance* **2014**, *239*, 44-49. DOI: 10.1016/j.jmr.2013.11.009.

(4) Earl, W. L.; Vanderhart, D. L. Measurement of ^{13}C chemical shifts in solids. *Journal of Magnetic Resonance* **1982**, *48* (1), 35-54. DOI: 10.1016/0022-2364(82)90236-0.

(5) Wilke, M.; Farges, F.; Petit, P.-E.; Brown, G. E.; Martin, F. Oxidation state and coordination of Fe in minerals: An FeK-XANES spectroscopic study. *American Mineralogist* **2001**, *86* (5-6), 714-730. DOI: 10.2138/am-2001-5-612.

(6) Hatcher, P. G.; Waggoner, D. C.; Chen, H. Evidence for the existence of humic acids in peat soils based on solid-state ^{13}C NMR. *Journal of Environmental Quality* **2019**, *48* (6), 1571-1577. DOI: 10.2134/jeq2019.02.0083.

(7) Sleighter, R. L.; McKee, G. A.; Liu, Z.; Hatcher, P. G. Naturally present fatty acids as internal calibrants for Fourier transform mass spectra of dissolved organic matter. *Limnology and Oceanography: Methods* **2008**, *6*, 246-253. DOI: 10.4319/lom.2008.6.246.

(8) Kujawinski, E. B.; Behn, M. D. Automated analysis of electrospray ionization Fourier transform ion cyclotron resonance mass spectra of natural organic matter. *Analytical chemistry* **2006**, *78* (13), 4363-4373. DOI: 10.1021/ac0600306.

(9) Koch, B. P.; Dittmar, T.; Witt, M.; Kattner, G. Fundamentals of molecular formula assignment to ultrahigh resolution mass data of natural organic matter. *Analytical chemistry* **2007**, *79* (4), 1758-1763. DOI: 10.1021/ac061949s.

(10) Stubbins, A.; Spencer, R. G. M.; Chen, H. M.; Hatcher, P. G.; Mopper, K.; Hernes, P. J.; Mwamba, V. L.; Mangangu, A. M.; Wabakanghanzi, J. N.; Six, J. Illuminated darkness: Molecular signatures of Congo River dissolved organic matter and its photochemical alteration as revealed by ultrahigh precision mass spectrometry. *Limnology and Oceanography* **2010**, *55* (4), 1467-1477. DOI: 10.4319/lo.2010.55.4.1467.

(11) Koch, B. P.; Dittmar, T. From mass to structure: An aromaticity index for high-resolution mass data of natural organic matter. *Rapid Communications in Mass Spectrometry* **2006**, *20* (5), 926-932. DOI: 10.1002/rcm.2386.

(12) Koch, B. P.; Dittmar, T. From mass to structure: An aromaticity index for high-resolution mass data of natural organic matter (Erratum). *Rapid Communications in Mass Spectrometry* **2016**, *30* (1), 250-250. DOI: 10.1002/rcm.7433.

(13) Osterholz, H.; Kirchman, D. L.; Niggemann, J.; Dittmar, T. Environmental drivers of dissolved organic matter molecular composition in the Delaware Estuary. *Frontiers in Earth Science* **2016**, *4*, 1-14. DOI: 10.3389/feart.2016.00095.

(14) Goranov, A. I.; Sleighter, R. L.; Yordanov, D. A.; Hatcher, P. TEnvR: MATLAB-based toolbox for environmental research. *Analytical Methods* **2023**, (15), 5390-5400. DOI: 10.1039/D3AY00750B.

(15) Stevenson, F. J. *Humus chemistry: Genesis, composition, reactions*; John Wiley & Sons, 1994.

(16) Guggenberger, G. Humification and mineralization in soils. In *Microorganisms in soils: Roles in genesis and functions*, Varma, A., Buscot, F. Eds.; Soil Biology, Springer, 2005; pp 85-106.

(17) Bostick, K. W.; Zimmerman, A. R.; Wozniak, A. S.; Mitra, S.; Hatcher, P. G. Production and composition of pyrogenic dissolved organic matter from a logical series of laboratory-generated chars. *Frontiers in Earth Science* **2018**, *6* (43), 1-14. DOI: 10.3389/feart.2018.00043.

(18) Simpson, A. J.; Simpson, M. J. Nuclear magnetic resonance analysis of natural organic matter. In *Biophysico-chemical processes involving natural nonliving organic matter in environmental systems*, Senesi, N., Xing, B., Huang, P. M. Eds.; 2009; pp 589-650.

(19) Mao, J.; Cao, X.; Olk, D. C.; Chu, W.; Schmidt-Rohr, K. Advanced solid-state NMR spectroscopy of natural organic matter. *Progress in Nuclear Magnetic Resonance Spectroscopy* **2017**, *100*, 17-51. DOI: 10.1016/j.pnmrs.2016.11.003.

(20) Gil, A. M.; Neto, C. P. Solid-state NMR studies of wood and other lignocellulosic materials. *Ann. R. NMR S.* **1999**, *37*, 75-117. DOI: 10.1016/S0066-4103(08)60014-9.

(21) Hedges, J. I.; Cowie, G. L.; Ertel, J. R.; Barbour, R. J.; Hatcher, P. G. Degradation of carbohydrates and lignins in buried woods. *Geochimica et Cosmochimica Acta* **1985**, *49* (3), 701-711. DOI: 10.1016/0016-7037(85)90165-6.

(22) Moody, G. J. The action of fenton's reagent on carbohydrates. *Tetrahedron* **1963**, *19* (11), 1705-1710. DOI: 10.1016/s0040-4020(01)99244-0.

(23) Morelli, R.; Russo-Volpe, S.; Bruno, N.; Lo Scalzo, R. Fenton-dependent damage to carbohydrates: free radical scavenging activity of some simple sugars. *Journal of agricultural and food chemistry* **2003**, *51* (25), 7418-7425. DOI: 10.1021/jf030172q.

(24) Pfeffer, P. E.; Gerasimowicz, W. V.; Piotrowski, E. G. Effect of paramagnetic iron on quantitation in carbon-13 cross polarization magic angle spinning nuclear magnetic resonance spectrometry of heterogeneous environmental matrixes. *Analytical chemistry* **2002**, *56* (4), 734-741. DOI: 10.1021/ac00268a032.

(25) Botto, R. E.; Wilson, R.; Winans, R. E. Evaluation of the reliability of solid ^{13}C NMR spectroscopy for the quantitative analysis of coals: Study of whole coals and maceral concentrates. *Energy & Fuels* **2002**, *1* (2), 173-181. DOI: 10.1021/ef00002a006.

(26) Hertkorn, N.; Ruecker, C.; Meringer, M.; Gugisch, R.; Frommberger, M.; Perdue, E. M.; Witt, M.; Schmitt-Kopplin, P. High-precision frequency measurements: Indispensable tools at the core of the molecular-level analysis of complex systems. *Analytical and Bioanalytical Chemistry* **2007**, *389* (5), 1311-1327. DOI: 10.1007/s00216-007-1577-4.

(27) Hertkorn, N.; Frommberger, M.; Witt, M.; Koch, B. P.; Schmitt-Kopplin, P.; Perdue, E. M. Natural organic matter and the event horizon of mass spectrometry. *Analytical chemistry* **2008**, *80* (23), 8908-8919. DOI: 10.1021/ac800464g.

(28) Zhang, X.; Han, J.; Zhang, X.; Shen, J.; Chen, Z.; Chu, W.; Kang, J.; Zhao, S.; Zhou, Y. Application of Fourier transform ion cyclotron resonance mass spectrometry to characterize natural organic matter. *Chemosphere* **2020**, *260*, 1-10. DOI: 10.1016/j.chemosphere.2020.127458.

(29) Sleighter, R. L.; Hatcher, P. G. The application of electrospray ionization coupled to ultrahigh resolution mass spectrometry for the molecular characterization of natural organic matter. *Journal of Mass Spectrometry* **2007**, *42* (5), 559-574. DOI: 10.1002/jms.1221.

(30) Hockaday, W. C.; Grannas, A. M.; Kim, S.; Hatcher, P. G. The transformation and mobility of charcoal in a fire-impacted watershed. *Geochimica et Cosmochimica Acta* **2007**, *71* (14), 3432-3445. DOI: 10.1016/j.gca.2007.02.023.

(31) Wagner, S.; Jaffe, R.; Stubbins, A. Dissolved black carbon in aquatic ecosystems. *Limnology and Oceanography Letters* **2018**, *3* (3), 168-185. DOI: 10.1002/lol2.10076.

(32) Stubbins, A.; Silva, L. M.; Dittmar, T.; Van Stan, J. T. Molecular and optical properties of tree-derived dissolved organic matter in throughfall and stemflow from live oaks and Eastern Red Cedar. *Frontiers in Earth Science* **2017**, *5*, 1-13. DOI: 10.3389/feart.2017.00022.

(33) Roth, V. N.; Lange, M.; Simon, C.; Hertkorn, N.; Bucher, S.; Goodall, T.; Griffiths, R. I.; Mellado-Vazquez, P. G.; Mommer, L.; Oram, N. J.; Weigelt, A.; Dittmar, T.; Gleixner, G. Persistence of dissolved organic matter explained by molecular changes during its passage through soil. *Nature Geoscience* **2019**, *12* (9), 755-761. DOI: 10.1038/s41561-019-0417-4.

(34) Wagner, S.; Dittmar, T.; Jaffe, R. Molecular characterization of dissolved black nitrogen via electrospray ionization Fourier transform ion cyclotron resonance mass spectrometry. *Organic Geochemistry* **2015**, *79*, 21-30. DOI: 10.1016/j.orggeochem.2014.12.002.

(35) Hertkorn, N.; Harir, M.; Cawley, K. M.; Schmitt-Kopplin, P.; Jaffe, R. Molecular characterization of dissolved organic matter from subtropical wetlands: A comparative study through the analysis of optical properties, NMR and FTICR/MS. *Biogeosciences* **2016**, *13* (8), 2257-2277. DOI: 10.5194/bg-13-2257-2016.

(36) Sleighter, R. L.; Chen, H.; Wozniak, A. S.; Willoughby, A. S.; Caricasole, P.; Hatcher, P. G. Establishing a measure of reproducibility of ultrahigh-resolution mass spectra for complex mixtures of natural organic matter. *Analytical chemistry* **2012**, *84* (21), 9184-9191. DOI: 10.1021/ac3018026.

(37) DiDonato, N.; Hatcher, P. G. Alicyclic carboxylic acids in soil humic acid as detected with ultrahigh resolution mass spectrometry and multi-dimensional NMR. *Organic Geochemistry* **2017**, *112*, 33-46. DOI: 10.1016/j.orggeochem.2017.06.010.

(38) DiDonato, N.; Chen, H. M.; Waggoner, D.; Hatcher, P. G. Potential origin and formation for molecular components of humic acids in soils. *Geochimica et Cosmochimica Acta* **2016**, *178*, 210-222. DOI: 10.1016/j.gca.2016.01.013.

(39) Waggoner, D. C.; Chen, H. M.; Willoughby, A. S.; Hatcher, P. G. Formation of black carbon-like and alicyclic aliphatic compounds by hydroxyl radical initiated degradation of lignin. *Organic Geochemistry* **2015**, *82*, 69-76. DOI: 10.1016/j.orggeochem.2015.02.007.

(40) Chen, H. M.; Abdulla, H. A. N.; Sanders, R. L.; Myneni, S. C. B.; Mopper, K.; Hatcher, P. G. Production of black carbon-like and aliphatic molecules from terrestrial dissolved organic matter in the presence of sunlight and iron. *Environmental Science & Technology Letters* **2014**, *1* (10), 399-404. DOI: 10.1021/ez5002598.

(41) Chang, Z.; Tian, L.; Li, F.; Zhou, Y.; Wu, M.; Steinberg, C. E. W.; Dong, X.; Pan, B.; Xing, B. Benzene polycarboxylic acid - A useful marker for condensed organic matter, but not for only pyrogenic black carbon. *Science of the Total Environment* **2018**, *626*, 660-667. DOI: 10.1016/j.scitotenv.2018.01.145.

(42) Gerke, J. Black (pyrogenic) carbon in soils and waters: A fragile data basis extensively interpreted. *Chemical and Biological Technologies in Agriculture* **2019**, *6* (13), 1-8. DOI: 10.1186/s40538-019-0151-6.

(43) Chen, X.; Ye, X.; Chu, W.; Olk, D. C.; Cao, X.; Schmidt-Rohr, K.; Zhang, L.; Thompson, M. L.; Mao, J.; Gao, H. Formation of char-like, fused-ring aromatic structures from a nonpyrogenic pathway during decomposition of wheat straw. *Journal of agricultural and food chemistry* **2020**, *68* (9), 2607-2614. DOI: 10.1021/acs.jafc.9b06037.

(44) Bates, A. L.; Hatcher, P. G. Quantitative solid-state ^{13}C nuclear magnetic resonance spectrometric analyses of wood xylen: Effect of increasing carbohydrate content. *Organic Geochemistry* **1992**, *18* (4), 407-416. DOI: 10.1016/0146-6380(92)90103-5.

(45) Waggoner, D. C.; Chen, H.; Willoughby, A. S.; Hatcher, P. G. Formation of black carbon-like and alicyclic aliphatic compounds by hydroxyl radical initiated degradation of lignin. *Organic Geochemistry* **2015**, *82*, 69-76. DOI: https://doi.org/10.1016/j.orggeochem.2015.02.007.

(46) DiDonato, N.; Chen, H.; Waggoner, D.; Hatcher, P. G. Potential origin and formation for molecular components of humic acids in soils. *Geochimica et Cosmochimica Acta* **2016**, *178*, 210-222. DOI: https://doi.org/10.1016/j.gca.2016.01.013.

(47) Waggoner, D. C.; Wozniak, A. S.; Cory, R. M.; Hatcher, P. G. The role of reactive oxygen species in the degradation of lignin derived dissolved organic matter. *Geochimica et Cosmochimica Acta* **2017**, *208*, 171-184. DOI: 10.1016/j.gca.2017.03.036.

(48) Zelinka, S.; Kirker, G.; Jakes, J.; Passarini, L.; Lai, B. Distribution and oxidation state of copper in the cell walls of treated wood examined by synchrotron based XANES and XFM. In *Corrosion Conference and Expo 2017*, New Orleans, LA, 2016; NACE International: pp 172-178.

(49) Zelinka, S. L.; Lakes, J. E.; Kirker, G. T.; Passarini, L.; Hunt, C. G.; Lai, B.; Antipova, O.; Li, L. X.; Vogt, S. Copper distribution and oxidation states near corroded fasteners in treated wood. *SN Applied Sciences* **2019**, *1* (3), 1-10. DOI: 10.1007/s42452-019-0249-2.

(50) Weng, Y.-T.; Rathod, J.; Liang, B.; Wang, C.-C.; Iizuka, Y.; Tamura, N.; Chen, C.-L.; Lee, Y.-C. Black carbon enriches short-range-order ferrihydrite in Amazonian Dark Earth: Interplay mechanism and environmental implications. *Science of the Total Environment* **2020**, *725*, 1-12. DOI: 10.1016/j.scitotenv.2020.138195.

(51) Trusiak, A.; Treibergs, L.; Kling, G.; Cory, R. The controls of iron and oxygen on hydroxyl radical (•OH) production in soils. *Soil Systems* **2018**, *3* (1), 1-23. DOI: 10.3390/soilsystems3010001.

(52) Pham, A. N.; Xing, G. W.; Miller, C. J.; Waite, T. D. Fenton-like copper redox chemistry revisited: Hydrogen peroxide and superoxide mediation of copper-catalyzed oxidant production. *Journal of Catalysis* **2013**, *301*, 54-64. DOI: 10.1016/j.jcat.2013.01.025.
